# Alpha 2A adrenergic receptor antagonism reduces fibrosis, inflammation and portal hypertension in models of chronic liver disease

**DOI:** 10.1016/j.jhepr.2026.101922

**Published:** 2026-06-09

**Authors:** Karen Louise Thomsen, Vikram Sharma, Helen Jones, Anne Catrine Daugaard Mikkelsen, Nathan Davies, Abeba Habtesion, Sara Ellegaard Paaske, Jane Macnaughtan, Maria Castanho Martins, Rocio Gallego-Durán, Manuel Romero-Gómez, Stephen Hamilton-Dutoit, Krista Rombouts, Rajiv Jalan, Rajeshwar Prosad Mookerjee

**Affiliations:** 1 UCL Institute of Liver and Digestive Health, University College London, United Kingdom; 2 Department of Hepatology & Gastroenterology, Aarhus University Hospital, Denmark; 3 SeLiver Group, Instituto de Biomedicina de Sevilla (HUVR/CSIC/US), Hepatic and Digestive Diseases Networking Biomedical Research Centre (CIBERehd), Digestive Diseases Unit, Hospital Universitario Virgen del Rocío, Department of Medicine, University of Seville, Sevilla, Spain; 4 Institute of Pathology, Aarhus University Hospital, Denmark

**Keywords:** Sympathetic signalling, cirrhosis, metabolic dysfunction-associated steatotic liver disease, steatohepatitis, noradrenaline, portal pressure

## Abstract

**Background & Aims:**

Sympathetic nervous system over-activity is associated with liver disease progression and development of portal hypertension, influencing systemic inflammation. This study examined the role of the sympathetic alpha-2A adrenergic receptor (ADRA2a) and its antagonism in hepatic stellate cell (HSC) activation, a key event in fibrosis progression, in experimental metabolic dysfunction-associated steatohepatitis (MASH) and in portal pressure elevation in a cirrhotic-rat model.

**Methods:**

An established bile duct ligation (BDL)-induced cirrhosis model (n = 14) was used to assess ADRA2a’s role in portal hypertension through acute treatment with two ADRA2A antagonists (BRL44408 and yohimbine). Human (h)HSCs were incubated with either an ADRA2a agonist (guanfacine) or antagonist (BRL44408), to determine whether ADRA2a modulation altered HSC activation. We also investigated ADRA2a expression in patients with MASH fibrosis (n = 15) and conducted a longer-term yohimbine treatment study in a diet-induced MASH rodent model (n = 24).

**Results:**

BRL44408 reduced portal pressure in BDL rats (12 ± 3 *vs.* 18 ± 4 mmHg; *p <*0.0001) while preserving mean arterial pressure (102 ± 16 *vs.* 93 ± 13 mmHg, *p* = 0.13). This was associated with (i) restored eNOS phosphorylation towards control levels and reduced caveolin-1 expression (*p <*0.05), and (ii) reduced hepatic inflammation and Kuppfer cell activation (*p <*0.001). Moreover, guanfacine stimulation of hHSCs increased their contractility, which was attenuated by BRL44408 (*p <*0.001). *ADRA2A* mRNA expression was increased in both patients with MASH fibrosis, and MASH rats. In MASH rats, yohimbine treatment reduced fibrosis, as measured by collagen proportional area (*p* <0.01).

**Conclusions:**

ADRA2A expression is increased in two experimental models of liver disease and in patients with MASH fibrosis, and it appears to contribute to the pathogenesis of portal hypertension and fibrogenesis. These findings suggest that ADRA2A antagonism may represent a potential therapeutic strategy for treating portal hypertension and fibrosis progression.

**Impact and implications:**

Managing fibrosis progression and portal hypertension remains a major challenge in liver disease, with fewer than 60% of patients responding to current non-selective beta-blocker therapy, despite clear evidence of increased sympathetic activation as liver disease advances. We show that the alpha-2A adrenergic receptor (ADRA2A) may represent an important pathway in hepatic stellate cell activation and may also influence additional mechanisms that regulate elevated portal pressure. These findings provide an alternative approach to beta-blockade for portal hypertension, which is limited by reductions in cardiac output and liver blood flow that can be problematic in patients with advanced disease. Data from two different rodent models support consideration of a translational clinical study of ADRA2A antagonism in portal hypertension and further investigation into the mechanisms by which ADRA2A antagonism affects metabolic dysfunction-associated steatotic liver disease.

## Highlights

• Hepatic ADRA2a expression increases in liver disease.
• Antagonists of ADRA2a decrease stellate cell activation in culture.
• ADRA2a antagonism reduces portal hypertension and fibrosis in liver disease models.
• ADRA2a antagonism may be a potential therapeutic target in evolving liver disease.

## Graphical abstract

**Figure:**
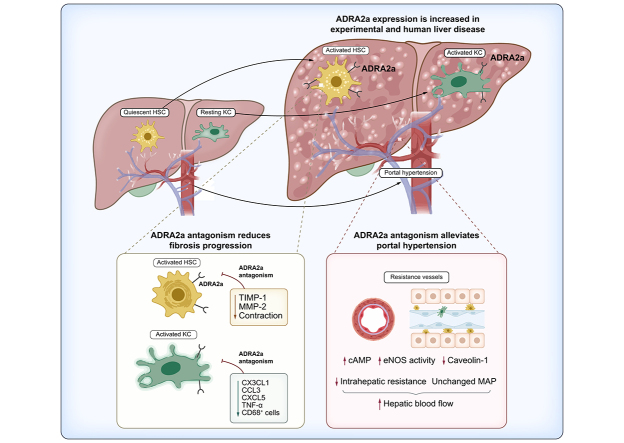

## Introduction

The pathophysiology of portal hypertension is well described,^1^ combining hepatic architectural changes, hepatic stellate cell (HSC) activation and endothelial dysfunction. Sympathetic nervous system overactivity is associated with liver disease progression and the development of portal hypertension; however, the limited response to portal pressure reduction with non-selective beta-blockers (NSBBs) (40–50%)^2^ highlights the need to consider other mechanistic targets.

Of interest, both steatosis and metabolic dysfunction-associated steatohepatitis (MASH) are associated with increasing portal pressure in animal models and patients, even in the absence of fibrosis progression.^3^ As MASH progresses, chronic inflammation and hepatocyte injury ultimately lead to fibrogenesis, associated with long-term morbidity and mortality.^4^ The main cell type responsible for extracellular matrix deposition is the HSC, which is also a key pathophysiological driver in the development of portal hypertension.^5^ Current licensed treatments for MASH, until recently, have largely been limited to modification of risk factors such as obesity and diabetes melitus.

The autonomic nervous system influences systemic inflammation.^6^ Levels of norepinephrine (NE) are elevated in acute decompensation of cirrhosis and correlate with increased intrahepatic resistance, changes in organ blood flow and severity of inflammation.^7^^,^^8^ NE metabolites have recently been linked with progression of acute cirrhosis decompensation.^9^ Moreover, animal models of sepsis suggest that NE participates in the development of hepatocellular dysfunction^6^^,^^10^ and that the gut and innate immune system are major autocrine sources of NE.^11^ NE-activated macrophages result in downstream generation of pro-inflammatory cytokines, further amplifying an inflammatory cascade,^3^ while also promoting and prolonging the activation of HSCs.^12^ Studies have suggested that increased NE levels in sepsis modulate the responsiveness of macrophages to pro-inflammatory stimuli, through increased expression of the alpha-2a adrenergic receptor (ADRA2a), in turn perpetuating inflammation^13^ and promoting hepatocellular dysfunction.^6^^,^^10^

ADRA2a is a member of the G-protein coupled receptor family and is abundant in many tissues, particularly within the reticuloendothelial system. On large arteries, ADRA2a maintains vessel tone, while in arterioles and venules, they paradoxically promote vasoconstriction.^14^^,^^15^ It follows that selective antagonism of ADRA2A may increase systemic blood pressure while also enhancing blood flow through resistance vessels. Selective antagonism of ADRA2a in sepsis models has been shown to decrease liver injury and improve outcomes.^16^

Given that *in vitro* NE stimulates HSCs to proliferate and increase extracellular matrix production,^17^^,^^18^ this raises the question of whether NE signals through ADRA2A expressed on HSCs and thereby influences fibrogenesis and portal hypertension. Therefore, this study aimed to examine the effects of ADRA2a on portal pressure in a rat model of severe portal hypertension; their role in promoting HSC activation; and finally, as to whether ADRA2a antagonism serves as a possible treatment for MASH in a diet-induced rat MASH model.

## Materials and methods

### ADRA2a antagonists

BRL44408 (Sigma-Aldrich, Dorset, UK), previously demonstrated to be a highly selectivity antagonist for the ADRA2a receptor sub-type,^19^ was used for proof of concept in target studies for BDL portal hypertension pressure lowering, and for *in vitro* challenge of HSCs.

Yohimbine hydrochloride (YoHCl) (Sigma-Aldrich) also has good pharmacological selectivity for ADRA2a with no beta or alpha 1 receptor activity, and has been used clinically in therapy for male impotence and adjunctive diabetic therapy.^20^ Given this, use of yohimbine repurposed for liver disease would be attractive if a beneficial effect was to be demonstrated.

### Animals

Male Sprague Dawley rats (body weight 220-300 g; Charles River, UK) were housed in a temperature and light controlled (12 h light/dark cycle) facility at University College London as described in more detail in the supplementary methods.

*BDL animal model*: Rats underwent bile duct ligation (BDL) as previously described.^21^ Sham-operated rats had a laparotomy without BDL. Four weeks after BDL or sham surgery, rats were allocated to one of four groups: (i) sham-operated (control) rats administered subcutaneous (sc) saline; (ii) BDL rats administered sc saline; (iii) BDL rats receiving sc BRL44408 (10 mg/kg, based on prior literature)^19^^,^^22^ administered at 24 h and again 3 h prior to haemodynamic measurements; and (iv) sham-operated rats administered sc BRL44408 prior to haemodynamic measurements. Similarly, a cohort of BDL rats were also administered YoHCl [0.4 mg/kg based on prior literature^23^ and our own dose ranging studies] for two doses, within 24 h of haemodynamic assessment as described in the supplemental methods.

*MASH animal model:* Twenty-four rats were fed normal chow (NC) (RM1; Special Diet Services, Witham, UK) or a high-fat, high-cholesterol (HFHC) diet (D09052204Y; Research Diets, USA) for 16 weeks as described previously.^24^ For the final 8 weeks of the study, HFHC-fed rats were randomly allocated to receive YoHCl in their drinking water or control. Fluid was adjusted to achieve an average dose of 0.4 mg/kg/day. The animal groups were: (i) NC group: NC for 16 weeks (n = 9), (ii) HFHC group: HFHC diet for 16 weeks (n = 6) and (iii) HFHC+YoHCl group: HFHC diet and YoHCl for a final 8 weeks of study (n = 9).

### Patients with MASLD

Fifteen patients with metabolic dysfunction-associated steatotic liver disease (MASLD) were recruited from the Virgen del Rocío University Hospital, Spain as described in the supplementary methods. Liver biopsies were obtained and mRNA expression of *ADRA2a* was evaluated using reverse-transcription quantitative PCR (RT-qPCR) as described in the supplementary methods.

### HSCs

#### Cell isolation, culture, treatment, gel contraction and RT-qPCR

Primary human hepatic stellate cells (hHSCs) were isolated from wedge resection sections of human healthy liver tissue as previously described,^25^ from patients undergoing surgery for primary liver cancer or metastasis from extrahepatic cancers at the Royal Free Hospital, London, UK after giving informed consent (REC reference^21^/WA/0388). Cells were isolated according to Mederacke *et al.*^26^ with modifications for human liver^27^ and cultured and treated with an ADRA2a agonist (guanfacine) or antagonist (BRL44408), as described in the supplementary methods. Gene expression of *ADRA2a* and *PDGFRβ* (platelet-derived growth factor receptor-β) in hHSCs was analysed by RT-qPCR, (described in supplemental methods).

### Blood analyses

Plasma concentrations of alanine aminotransferase (ALT), aspartate aminotransferase (AST), albumin, bilirubin, and total cholesterol were measured using a Cobas Integra 400 automated analyser (Roche Diagnostics Ltd., Burgess Hill, UK).

#### Norepinephrine

Norepinephrine (NE) was extracted from plasma samples (EDTA), standards, and controls using a boronate affinity gel-coated plate. The extracted NE was acylated and enzymatically derivatised. The concentration of derivatised NE was measured using a competitive ELISA (LDN Labor Diagnostika Nord GmbH & Co. KG, Nordhorn, Germany) with a HRP-conjugated secondary antibody and TMB as substrate, and measured via a 96-well plate reader (BMG Labtech Ltd., UK).

#### FACS characterization of monocytes

The monocyte phagocytic-capacity was examined using FACS (described in the supplementary methods).

### Liver tissue analyses

#### Liver histology

Formalin fixed liver tissue was processed and embedded in paraffin. 3-5 mm sections were de-waxed in xylene, stained with H&E or picrosirius red (PSR), and mounted in DPX. Slides were digitised using a NanoZoomer 2.0-HT digital slide scanner (Hamamatsu Photonics UK Ltd, Welwyn Garden City, UK). All histology specimens from MASH animals were reviewed by an experienced liver histopathologist (SHD) blinded to the clinical and laboratory data. NAFLD activity score was assessed using the Kleiner (NASH CRN) system.^28^

#### Collagen proportionate area measurement

Collagen proportionate area (CPA) was determined on NanoZoomer images of PSR-stained whole sections of liver as described in the supplementary methods.

#### Liver fat content quantification

Liver fat content was quantified using an adipogenesis detection assay kit (Abcam plc., UK) according to the manufacturer’s instructions. Briefly, lipids were extracted from accurately weighed liver tissues, hydrolysed to free fatty acids and glycerol, and the released glycerol oxidised to generate colour from the probe, measured using a 96-well plate reader (BMG Labtech Ltd.).

#### Cyclic AMP analyses

Cyclic AMP in liver tissue was analysed using an ELISA technique. This was done by using 96-well plates pre-coated with mouse monoclonal anti-rabbit IgG, and blocked with a proprietary formulation of proteins (Cayman Chemical, Ann Arbor, MI, USA).

#### Western blot analysis

In BDL animals, hepatic protein expression of ADRA2a, caveolin-1, endothelial nitric oxide synthase (eNOS) and p-eNOS was measured using WB analysis as previously described^29^ and in the supplementary methods.

#### RNA isolation and RT-qPCR

In MASH animals, mRNA expression of *ADRA2a* and *TIMP1* (TIMP metallopeptidase inhibitor 1) was determined using RT-qPCR (details in supplemental methods).

### Hepatic MMP-2 protein expression

Protein expression of active matrix metalloproteinase-2 (MMP-2) in MASH animals was measured using gel zymography as described in the supplemental methods.

### Hepatic protein expression of chemokines

In MASH animals, a rat cytokine and chemokine proteome profiler array (ARY008; Bio-Techne Ltd, Abingdon, UK) was used to screen 29 analytes, to determine the most relevant to be measured by ELISA. Hepatic protein expression of Ccl3 (macrophage inflammatory protein [MIP]-1α), Cx3cl1 (fractalkine) and Cxcl5 (LPS-induced CXC chemokine [LIX]) was then assessed by ELISA as described in the supplementary methods.

### Hepatic CD68 protein expression

Liver sections (3–5 mm) from MASH animals were dewaxed in xylene and rehydrated through graded alcohols, and CD68 was detected by immunohistochemistry (IHC) as described in the supplementary methods.

#### Kupffer cell phagocytic function and ROS production

Isolation of liver non-parenchymal cells, the Kupffer cell phagocytic function and their reactive oxygen species (ROS) production were assessed as described in the supplementary methods.

### Statistical analysis

Statistical analysis was performed using Prism 6 (GraphPad Software Inc., San Diego, CA, USA). The assumption of normality was checked using quantile-quantile plots (Q-Q plots) and histograms to ensure normally distributed variables. Differences between normally distributed variables were evaluated by ANOVA; when significant, *post hoc* tests were performed among groups by Student’s *t* test. Some variables showed skewed distributions with variance heterogeneity and were analysed using Kruskal-Wallis rank test. When significant, *post hoc* tests were performed among groups by the Mann-Whitney test. Normally distributed data are presented as mean ± SD and non-parametric tested data are presented as median (IQR). *p* values <0.05 were considered statistically significant.

## Results

### ADRA2a plays a role in portal hypertension and hepatic inflammation

#### Study of a BDL animal model of significant portal hypertension

Body weight and liver biochemical parameters at termination are shown in Table 1. Following 24-hours treatment with the highly selective ADRA2a antagonist, BRL44408, there were no statistically significant changes in liver biochemical parameters. Evidence of chronic liver injury consistent with advanced fibrosis and nodule formation was demonstrated in the BDL rats compared with sham rats seen on H&E staining, as we have previously demonstrated^30^ and no discernible histological changes in fibrosis were observed after BRL44408 treatment.

**Table 1:** Body weights and biochemical characteristics.

	Sham	BDL	BDL + BRL	*p* value, sham *vs.* BDL
Whole BW, end (g)	429 ± 62	391 ± 53	381 ± 48	0.11
ALT (U/L)	70 ± 9	100 ± 13	82 ± 6	<0.01
AST (U/L)	131 ± 17	445 ± 58	406 ± 65	<0.01
Albumin (g/L)	32.4 ± 0.8	24.4 ± 0.9	25.7 ± 1.0	<0.01
Bilirubin (μmol/L)	44 ± 15	132 ± 21	123 ± 12	<0.01

Whole BW and plasma concentrations of ALT, AST, albumin and bilirubin in sham animals, BDL animals and BDL animals treated with BRL44408 (BRL). Sham and BDL groups were compared using Student’s *t*-test post-hoc after findig significant differences (*p <*0.05) comparing all three groups by one-way analysis of variance (ANOVA). Data are presented as mean ± SD. ALT, alanine aminotransferase; AST, aspartate aminotransferase; BDL, bile duct ligation; BW, body weight; BRL, BRL44408.

#### Hepatic expression of ADRA2a is increased in BDL cirrhosis

**Fig. 1:**
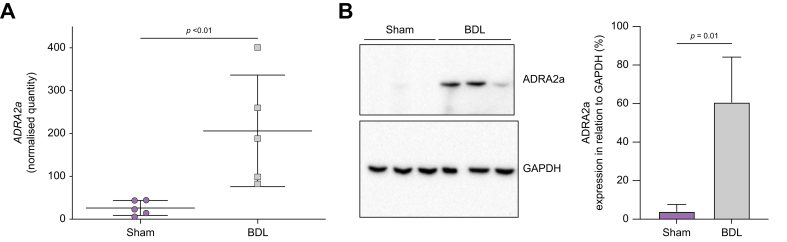
Hepatic expression of ADRA2a. Sham rat livers have barely detectable expression of *ADRA2a* at mRNA (A) and protein levels (B). BDL surgery in rats after 4 weeks induces a marked increase in expression of ADRA2a. mRNA and protein expression of ADRA2a is normalized to GAPDH. Sham and BDL groups were compared using the Mann-Whitney test. BDL, bile duct ligation.

Expression of ADRA2A at both mRNA and protein levels was significantly increased in BDL rats compared with sham animals (*p <*0.01) (Fig. 1).

#### Haemodynamic derangement in BDL cirrhotic rats is reversed by ADRA2a antagonism

**Fig. 2:**
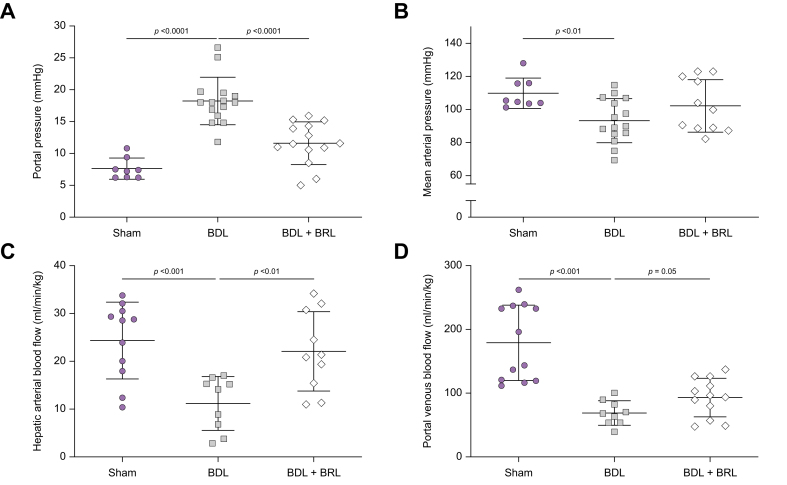
Haemodynamics. Portal pressure is markedly increased in BDL rats compared to sham and ADRA2a antagonism with BRL44408 (BRL) significantly lowers portal pressure in BDL animals (A). Mean arterial blood pressure is significantly reduced in BDL rats and this is restored back towards sham levels with antagonism of ADRA2a (B). Hepatic arterial blood flow (C) and portal venous flow (D) is significantly reduced in BDL rats compared to sham and there is a significant increase in hepatic arterial flow and a modest increase in portal venous flow after ADRA2a antagonism. Differences between groups were analysed using Kruskal-Wallis rank test. When significant, *post hoc* tests were performed among groups by the Mann-Whitney test. BDL, bile duct ligation; BRL, BRL44408.

As previously demonstrated,^30^ BDL rats demonstrated a marked increase in portal pressure compared to sham-operated rats (18 ± 4 *vs.* 8 ± 2 mmHg; *p <*0.0001), which was significantly reduced by BRL44408 (12 ± 3 mmHg; *p <*0.0001) (Fig. 2A). Moreover, the BDL rats had lower mean arterial pressure (93 ± 13 *vs.* 110 ± 9 mmHg; *p <*0.01), which tended to increase in BDL rats post ADRA2a antagonism (102 ± 16 mmHg; *p =* 0.13) (Fig. 2B). The hepatic artery and portal venous blood flows were both reduced in the BDL rats when compared to sham animals (11 ± 6 *vs.* 24 ± 8 ml/min/kg BW; *p <*0.001 and 69 ± 19 *vs.* 179 ± 59 ml/min/kg BW; *p <*0.0001, respectively), and BRL44408 promoted a significant increase in both hepatic arterial blood flow (22 ± 8 ml/min/kg BW, *p <*0.01) and portal venous blood flow (93 ± 30 ml/min/kg BW; *p =* 0.05) (Fig. 2C,D).

We validated our findings of acute portal pressure reduction via ADRA2A antagonism using a second intervention, YoHCl, given its potential for direct clinical translation. BDL rats treated with YoHCl within 24 h also had a significant lowering of portal pressure compared to BDL rats injected with saline (10 ± 1 *vs.* 15 ± 2 mmHg, *p <*0.01).

#### Sympathetic nervous system activation in BDL rats

**Fig. 3:**
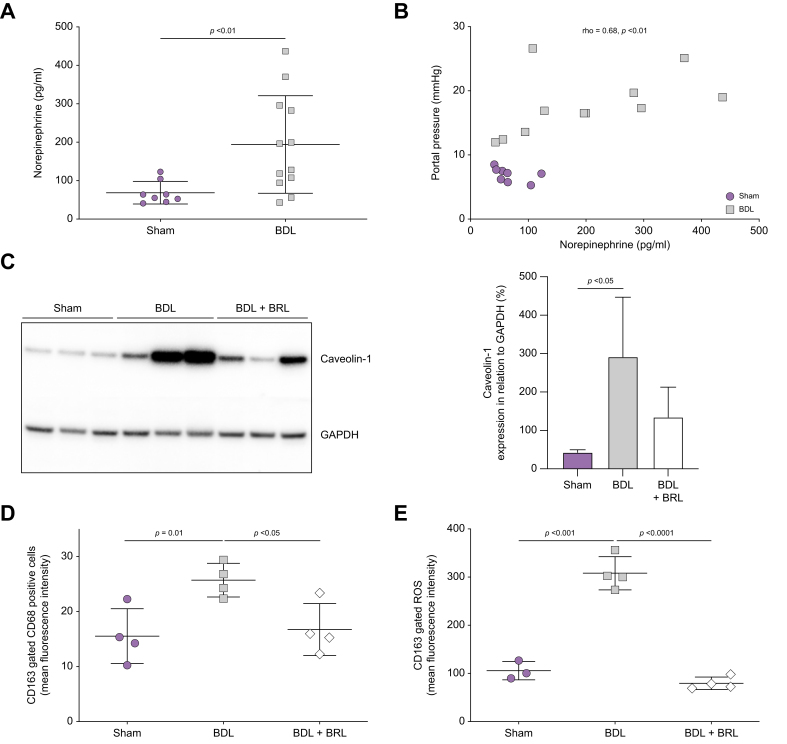
Sympathetic nervous system, caveolin-1 and markers of inflammation. Norepinephrine levels are markedly elevated in BDL rats compared to sham (A) (the Mann-Whitney test) and correlate with increasing portal pressure (B) (Pearson correlation test). Hepatic expression of caveolin-1, a known inhibitor of eNOS activity is increased in BDL rats compared to sham and reduced after BRL treatment (C) (the Mann-Whitney test). In liver tissue, numbers of activated Kupffer cells were increased in BDL animals compared to sham animals and markedly reduced following BRL treatment (D) (one-way ANOVA and Student’s *t* test). The generation of ROS by Kupffer cells also increased significantly in the BDL rats compared to sham and was reduced following BRL44408 treatment (E) (one-way ANOVA and Student’s *t* test). BDL, bile duct ligation; BRL, BRL44408; ROS, reactive oxygen species.

NE levels were markedly elevated in BDL rats compared to sham rats (194 ± 127 *vs.* 69 ± 29 pg/mL; *p <*0.01) (Fig. 3A). Furthermore, plasma NE was shown to correlate with increasing portal pressure (Pearson’s r = 0.68; *p <*0.001) (Fig. 3B).

#### Changes in cyclic AMP levels in BDL rats reflecting NE signaling through ADRA2a

cAMP levels tended to decrease in BDL rats compared to sham rats (276.6 ± 16.5 *vs.* 318.7 ± 16.9 nmol/L; *p =* 0.10). However, after BRL44408, treatment of BDL rats, cAMP levels increased compared with BDL rats treated with saline (340.3 ± 15.8 *vs.* 276.6 ± 16.5 nmol/L; *p =* 0.01).

#### Impaired eNOS activity in cirrhosis is ameliorated by ADRA2a antagonism

eNOS activity was assessed by the p-eNOS/eNOS ratio. eNOS activity was decreased in BDL compared to sham rats, as demonstrated by a p-eNOS/eNOS ratio of 0.022 ± 0.007 *vs.* 0.710 ± 0.045. However, there was a restoration of eNOS phosphorylation towards sham levels in BRL44408-treated BDL rats, demonstrated by a p-eNOS/eNOS ratio of 0.373 ± 0.051 compared to untreated BDL rats. Furthermore, expression of caveolin-1, a known inhibitor of eNOS activity, was increased in BDL compared to sham rats, and was commensurately reduced after ADRA2a antagonism (Fig. 3C).

#### Markers of systemic and liver inflammation are decreased by ADRA2a antagonism

A detailed FACS characterization of monocytes revealed that the monocyte phagocytic-capacity was reduced in BDL rats compared with sham animals (31.3 ± 4.4 *vs.* 73.4 ± 4.8 mean fluorescence intensity [MFI]; *p <*0.001) and was restored towards normal levels in the BDL rats after treatment with BRL44408 (60.7 ± 6.3 MFI; *p =* 0.03). Moreover, in homogenized liver tissue, CD163/CD68 gating was used to define an activated Kupffer cell population, which was increased in BDL animals compared with sham animals (25.7 ± 1.5 *vs.* 15.5 ± 2.5 MFI; *p =* 0.01) (Fig. 3D). Following treatment with BRL44408, the number of active Kupffer cells in BDL rats was markedly reduced (16.7 ± 2.4 MFI; *p <*0.05). Aligned with the increase in activation status of Kupffer cells, generation of ROS by Kupffer cells also increased significantly in the BDL compared to sham rats (308.0 ± 17.3 *vs.* 105.8 ± 11.01 MFI; *p <*0.001) and was reduced in BDL rats following treatment with BRL44408 (79.6 ± 6.5 MFI; *p <*0.001) (Fig. 3E).

Liver tissue TNFα protein expression was markedly increased in BDL rats compared with sham rats (149.5 ± 73.7 *vs.* 55.3 ± 16.8 pg/ml; *p <*0.01); this was significantly decreased after BRL44408 therapy (93.1 ± 16.3 pg/ml; *p =* 0.03). Similarly, in portal venous blood, TNFα expression was increased in BDL rats (48.1 ± 4.1 *vs.* 20.1 ± 6.3 pg/ml; *p <*0.01) and reduced by BRL44408 treatment (31.1 ± 5.4 pg/ml; *p =* 0.03). Liver IL-6 expression was also markedly reduced after BRL44408 treatment compared with untreated BDL animals (14.4 ± 6.5 *vs.* 77.2 ± 14.8 pg/ml; *p <*0.01).

### Isolated HSCs express ADRA2a and respond to ADRA2a agonism and antagonism

**Fig. 4:**
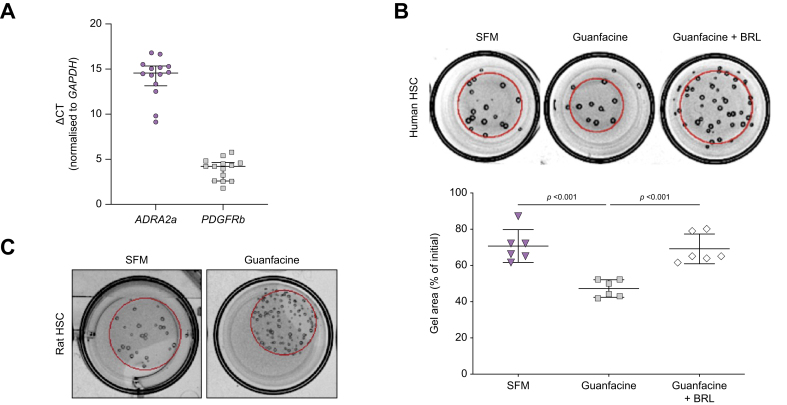
Expression of ADRA2a and contractility of HSCs. Human HSCs express both ADRA2a and PDGFRβ to varying levels amongst the 14 donor livers tested (A). Human HSCs grown in the presence of guanfacine show significantly greater contraction in a 3D collagen gel than cells grown in SFM in the absence of guanfacine. Cells grown in the presence of both guanfacine and BRL44408 show less contraction in a 3D collagen gel than cells grown in the presence of guanfacine alone (B) (one-way ANOVA and Student’s *t* test). Rat HSCs grown in the presence of guanfacine show greater contraction in a 3D collagen gel than cells grown in the absence of guanfacine (C). Of note, the black dots in the gel contraction areas represent artefacts (air bubbles). BRL, BRL44408; HSCs, hepatic stellate cells; SFM, serum-free medium.

We determined whether hHSCs express ADRA2a by performing qRT-PCR on hHSCs obtained from 14 liver donors. We found detectable *ADRA2a* transcripts at varying levels in the different cell isolates. *PDGFRβ*, a transcript known to be expressed by hHSCs, was also confirmed to be expressed in all tested donors (Fig. 4A). To determine whether ADRA2a expression could lead to functional HSC activation as demonstrated by hHSC contractility, hHSCs were incubated in serum-free medium (SFM), SFM with an ADRA2a agonist (guanfacine), or SFM with guanfacine and the ADRA2a antagonist, BRL44408. Representative images from the cell collagen gel contraction assay and its quantification are shown in Fig 4B. Stimulation of the hHSCs with guanfacine was associated with a significant increase in gel contraction compared to SFM (47 ± 5 % *vs.* 71 ± 9; *p <*0.001), while the addition of the antagonist BRL44408 significantly attenuated guanfacine-induced HSC contraction (69 ± 3 %; *p <*0.001).

To determine whether these observations in hHSCs could also induce HSC contraction in rat liver, rat HSCs were incubated in SFM or guanfacine. Stimulation with guanfacine also led to an increase in the contraction of the 3D collagen gel compared to SFM, as was also seen with hHSCs (Fig. 4C).

### ADRA2a expression is increased in patients with MASLD and evolving fibrosis

We investigated whether hepatic ADRA2a expression was increased in patients with MASLD fibrosis. In patients with MASLD and established F1-3 fibrosis, the hepatic mRNA expression of *ADRA2a* was increased compared to that in patients with F0 fibrosis (90.9 ± 81.6 *vs.* 19.4 ± 16.4). The clinical, biochemical, and histological characteristics of the two groups are shown in Table S1.

### Antagonism of ADRA2a decreases fibrosis and inflammation in MASH

#### Study of a MASH animal model of fibrosis

Table 2 describes the clinical, biochemical and histological differences between HFHC-fed animals and those on NC, as well as the impact of ADRA2a antagonism. No differences in body weight increments were observed between the groups after 16 weeks. However, over the final 8 weeks of intervention, HFHC-fed rats gained less weight than NC-fed animals. By contrast, YoHCl-treated animals on a HFHC diet had growth not dissimilar to NC*.* Of note, there were no differences in the kilo calories consumed between the three groups.

**Table 2:** Weights, biochemical characteristics and histological scores of liver tissue.

	Normal chow	HFHC diet	HFHC diet + YoHCl	*p* value	*p* value	*p* value
	n = 9	n = 6	n = 9	All 3 groups	NC *vs.* HFHC	HFHC *vs.* HFHC+Yo
Whole BW, start (g)	305 ± 26	309 ± 14	305 ± 16	0.91		
Whole BW, end (g)	591 ± 31	559 ± 41	581 ± 49	0.33		
Weight gain (g)	286 ± 31	250 ± 39	276 ± 38	0.16		
Weight gain (g) final 8 weeks	89 ± 17	53 ± 24	79 ± 20	0.01	<0.01	0.03
Liver weight (g)	16.4 ± 2.0	36.3 ± 5.2	33.5 ± 3.3	<0.0001	<0.0001	0.22
Liver/body weight (%)	2.8 ± 0.3	6.5 ± 0.9	5.8 ± 0.4	<0.0001	<0.0001	<0.05
Liver fat (μmol/mg liver)	17 (12-22)	41 (26-90)	29 (23-40)	<0.01	<0.01	0.39
Total cholesterol (mmol/L)	1.2 (1.1-1.5)	3.7 (2.7-4.5)	2.8 (2.4-3.5)	<0.001	<0.001	0.24
ALT (U/L)	40 (37-50)	142 (96-190)	113 (87-162)	<0.001	<0.001	0.49
AST (U/L)	100 (92-129)	329 (312-356)	260 (230-292)	0.0001	<0.001	0.02
Steatosis	0 ± 0	3 ± 3	3 ± 3	<0.0001	<0.0001	1.0
Inflammation	0.1 ± 0.3	1.0 ± 0.0	0.8 ± 0.8	0.01	0.001	0.53
Ballooning	0 ± 0	1.8 ± 0.4	0.6 ± 0.5	<0.0001	0.001	<0.01
NAFLD activity score	0.1 ± 0.3	5.8 ± 0.4	4.3 ± 1.1	<0.0001	0.001	<0.01
Fibrosis	0 ± 0	2.6 ± 1.1	1.1 ± 0.9	<0.0001	<0.0001	0.02

Whole BW, weight gain, and liver weight, liver fat concentration and plasma concentrations of total cholesterol, ALT, and AST and histological scores of liver tissue (steatosis, inflammation, ballooning, NAFLD activity score and fibrosis) in control animals (n = 9), HFHC-fed animals (n = 6) and HFHC-fed animals treated with YoHCl for the final 8 weeks of the 16-week study (n = 9). Normally distributed variables were analysed using one-way ANOVA and data showing skewed distributions and variance heterogeneity with Kruskal-Wallis rank test. When significant, *post hoc* tests were performed among groups by Student’s *t* test and the Mann-Whitney test, respectively. Data tested using parametric tests are presented as mean ± SD. Data tested using non-parametric tests are presented as median (IQR). ALT, alanine aminotransferase; AST, aspartate aminotransferase; BDL, bile duct ligation; BW, body weight; HFHC, high-fat high-cholesterol; YoHCl, yohimbine hydrochloride.

HFHC-fed rats had increased liver weight and a significantly higher liver-to-body weight ratio than NC-fed rats; both measures were significantly reduced by YoHCl treatment compared with untreated HFHC-fed rats. Plasma cholesterol, ALT and AST levels were increased in HFHC- compared to NC-fed rats, and YoHCl treatment significantly reduced AST levels but had less impact on ALT.

Microscopically, HFHC rat livers showed severe steatosis, with varying degrees of hepatocyte ballooning, inflammation and fibrosis. Overall, the HFHC-fed animals showed histological evidence of MASH; HFHC livers showed areas of perisinusoidal, periportal and bridging fibrosis. YoHCl treatment decreased the NAFLD activity score and degree of ballooning, in addition to reducing histological features of fibrosis compared to non-treated HFHC-fed rats (1.1 ± 0.9 *vs.* 2.6 ± 1.1; *p* = 0.02). As expected, livers from NC-fed animals had normal histology.

Liver fat content was quantified by measuring the mono-, di-, and triglycerides extracted from liver tissue. Liver fat content was significantly higher in HFHC-fed rats than NC-fed rats, and did not change significantly following YoHCl therapy (Table 2).

#### ADRA2a expression is increased in MASH rats

ADRA2a was significantly overexpressed in the livers of HFHC- compared to NC-fed animals (90.9 ± 33.2 *vs.* 36.6 ± 25.1; *p* = 0.001), and this expression was not changed by YoHCl treatment (73.3 ± 23.3; *p* = 0.23).

#### ADRA2a antagonism reduces progression of hepatic fibrosis in MASH rats

**Fig. 5:**
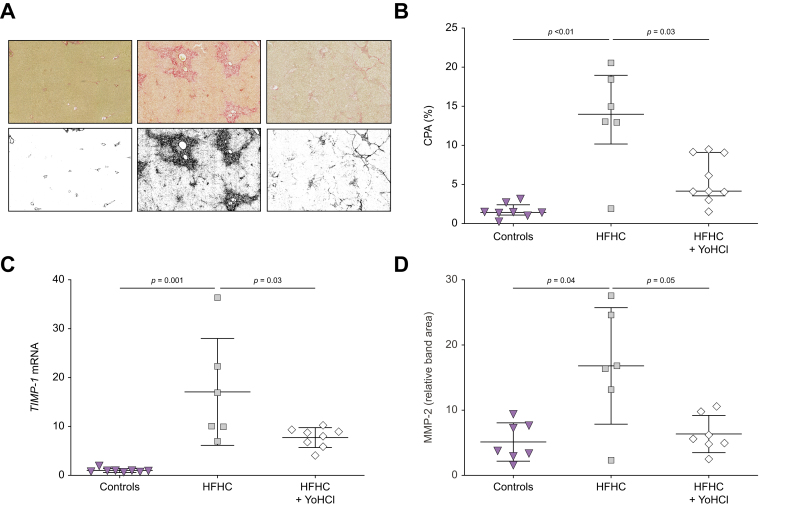
Hepatic fibrosis in MASH rats. CPA is increased in HFHC-induced MASH animals compared to controls and significantly reduced by YoHCl treatment, as shown by representative picrosirius red staining and CPA quantification of the liver (A, B). Hepatic gene expression *TIMP1* (C) and protein expression of active MMP-2 (D) are increased in HFHC-fed animals and significantly reduced by YoHCl. Differences between groups were analysed using Kruskal-Wallis rank test. When significant, *post hoc* tests were performed among groups by the Mann-Whitney test. CPA, collagen proportionate area; HFHC, high-fat high-cholesterol; MASH, metabolic dysfunction-associated steatohepatitis; YoHCl, yohimbine hydrochloride.

To quantify degree of fibrosis, CPA was measured by digital image analysis of PSR staining, as shown in Fig. 5A. CPA increased in HFHC-fed compared to NC-fed animals (15.0 (13.0-19.5) *vs.* 1.4 (0.6-1.5), *p* <0.01) and YoHCl treatment significantly reduced this (7.6 (4.1-9.2); *p* <0.01) (Fig. 5B).

The gene expression of TIMP1, the main regulator of extracellular matrix composition, was increased in HFHC- compared to NC-fed animals and significantly reduced by YoHCl (8.4 (6.1-9.2) *vs.* 13.5 (9.2-25.8), *p =* 0.03) (Fig. 5C). However, as fibrogenesis involves rearrangement as well as synthesis of collagen fibres, we also assessed MMP activity, as demonstrated by active MMP-2. Protein expression of active MMP-2 increased significantly in HFHC- compared to NC-fed animals (16.6 (10.5-25.3) *vs.* 3.8 (2.9-7.6); *p =* 0.04) and was reduced by YoHCl (5.6 (4.8-9.8), *p* = 0.05) (Fig. 5D), supporting the reduction in fibrosis seen on CPA analysis.

#### ADRA2a antagonism reduces hepatic inflammation in MASH rats

**Fig. 6:**
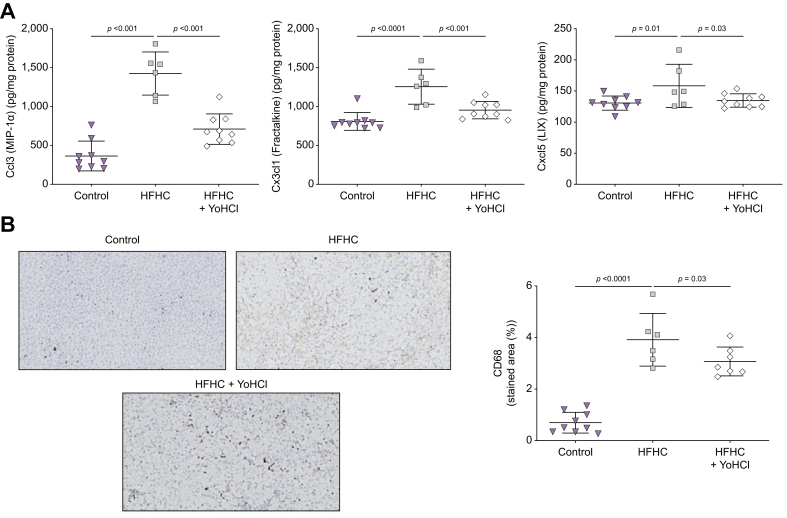
Hepatic inflammation in MASH rats. The increased hepatic protein expression of Ccl3 (macrophage inflammatory protein [MIP]-1α), Cx3cl1 (fractalkine) and Cxcl5 (LPS-induced CXC chemokine [LIX]) in HFHC-induced MASH rats are reduced by YoHCl (A). Likewise, YoHCl reduces the increased number of CD68-positive cells in the liver tissue. A representative CD68 immunohistochemical stain of liver tissue is shown (B). Differences between groups were analysed using Kruskal-Wallis rank test. When significant, *post hoc* tests were performed among groups by the Mann-Whitney test. HFHC, high-fat high-cholesterol; MASH, metabolic dysfunction-associated steatohepatitis; YoHCl, yohimbine hydrochloride.

To determine the most relevant cytokines and/or chemokines to investigate in HFHC rat liver, a pilot study using a proteome profiler array was performed (data not shown). This identified three cytokines and chemokines that were selected for further study: Ccl3 (MIP-1α), Cx3cl1 (fractalkine) and Cxcl5 (LIX). Protein expression of Ccl3 was increased in HFHC- compared to NC-fed animals (1,425 ± 278 *vs.* 365 ± 192; *p <*0.0001) and reduced by YoHCl (711 ± 196; *p <*0.0001). Similarly, protein expression of Cx3cl1 was increased in HFHC- compared to NC-fed animals (1256 ± 225 *vs.* 809 ± 114; *p <*0.0001) and reduced by YoHCl (955 ± 112; *p <*0.001). Finally, Cxcl5 protein expression was also increased in HFHC- compared to NC-fed animals (158 ± 35 *vs.* 131 ± 11); *p =* 0.01) and reduced by YoHCl (135 ± 11; *p =* 0.03) (Fig. 6A).

Moreover, in liver tissue, CD68 staining demonstrated increased numbers of resident macrophages and infiltrating monocytes/macrophages in HFHC- compared to NC-fed animals (3.92 ± 1.02 *vs.* 0.70 ± 0.40, *p <*0.0001). Treatment with YoHCl resulted in decreased numbers of CD68-positive cells (3.07 ± 0.56, *p =* 0.03) (Fig. 6B), supporting the reduction in hepatic inflammation.

## Discussion

In this study, we show that severity of portal hypertension in BDL cirrhotic rats correlates with NE concentrations and an increased expression of ADRA2a, whilst treatment with two different ADRA2a antagonists, namely BRL44408 and YoHCl, significantly lowers portal pressure. In addition, we show increased liver blood flow and preserved mean arterial pressure, suggesting a likely impact on intrahepatic resistance and an improvement in overall systemic haemodynamics with ADRA2a antagonism. This contrasts with current clinical treatment with beta-blockade which often impacts on the systemic circulation, lowering mean arterial pressure and liver blood flow, which may aggravate cirrhosis decompensation.^31^

Sympathetic overactivation has been associated with the progression of cirrhosis to decompensation,^32^ while plasma NE levels have been reported to correlate with the severity of portal hypertension, systemic inflammation, and clinical outcomes in cirrhosis.^8^^,^^9^^,^^33^ Increased sympathetic tone (NE), along with reduced vasodilatory factors, drives an increase in intrahepatic resistance in cirrhosis, compounded further by inflammation as cirrhosis progresses.^1^^,^^34^ At a mechanistic level, activation and contraction of HSCs, often in the context of hepatic inflammation, in conjunction with reduced liver generation of endothelial NO, are key elements behind an increase in hepatic vascular resistance.[35], [36], [37]

Given this, we assessed the impact on hepatic NO bioavailability and demonstrated that ADRA2a antagonism helps restore eNOS activity in BDL cirrhosis rats and reduces caveolin-1 expression (an inhibitor of eNOS). In addition, we show that inhibiting ADRA2a lowers systemic and hepatic inflammation, with reduced ROS generation and activation of Kupffer cells. This may, in part, explain the rapid reduction in portal pressure within 24 h post both BRL44408 and YoHCl treatment of BDL rats, as noted with other interventions that reduce eNOS inhibition and lower hepatic inflammation.^38^^,^^39^

To date, treatment of portal hypertension focusses largely on NSBBs to induce portal pressure reduction in about 40-50% of cases, largely by reducing portal inflow. However, NSBBs are associated with reduction in cardiac output from action on cardiac beta-1 receptors and reduced organ perfusion (including hepatic), which is most marked as cirrhosis progresses. More recently, agents such as carvedilol, also combining alpha 1-adrenergic receptor antagonism with beta-blockade, enhance portal pressure lowering but again negatively impact systemic haemodynamics. ADRA2a subtype receptors are reported to have distinct effects: their central activation decreases systemic blood pressure, in part by promoting NO release,^14^ whereas in small resistance vessels they induce vasoconstriction.^15^ This provided the rationale for antagonizing ADRA2a in the context of portal hypertension: to increase systemic blood pressure and improve blood flow through resistance vessels. These effects were indeed observed in our BDL cirrhosis model, where both hepatic arterial and portal venous components of liver blood flow improved following treatment with the highly selective ADRA2a antagonist BRL44408, which also reduced portal pressure while maintaining mean arterial pressure.

HSCs become activated during liver injury, such as in MASH and alcohol-related steatohepatitis, and undergo myofibroblastic transformation, whereby contractile elements modulate the tonicity of neighboring liver resistance vasculature.^35^ Inflammatory signals from activated innate immune cells, in addition to hepatocyte factors generated during liver injury, promote HSC activation.^40^^,^^41^ Moreover, activated HSCs in turn produce pro-fibrogenic factors and inflammatory cytokines, further adding to liver injury progression.^42^ In this study, we demonstrate increased activation of HSCs in patients and in rat primary HSCs upon stimulation with a selective ADRA2a agonist, guanfacine, which can be reversed by the addition of the highly selective ADRA2a antagonist BRL44408, with a functional reduction in contractility. This may provide a further mechanistic explanation for the reduction in portal pressure in BDL rats after ADRA2a antagonism.

In addition to its contribution to portal hypertension, HSC activation plays a key role in fibrogenesis. *In vitro*, adrenergic receptor antagonism reduces extracellular matrix production in a mouse HSC cell line^43^ and inhibits proliferation in mouse and rat primary HSCs.^17^ Moreover, chemical sympathectomy in a carbon tetrachloride rat model, ameliorates liver fibrogenesis, highlighting the importance of noradrenergic signaling in the development of fibrosis.^44^ In this study, reducing HSC contractility with ADRA2a antagonism is associated with improved cAMP generation, reflecting downstream signaling through the G-protein αi-subunit of ADRA2a.^45^ How NE acts on HSCs and contributes to liver disease progression has not been extensively explored to date; however, our data suggest a potential mechanism involving ADRA2a, as evidenced by *in vitro* effects on HSC activation, where selective ADRA2a antagonism decreased fibrogenic markers (TIMP-1 and MMP2 activity) and reduced CPA in our MASH model after 8 weeks of YoHCl treatment. This requires further elaboration in other fibrosis models.

ADRA2a is expressed in high concentrations in the mesenteric vascular bed and on immune cells, particularly in the reticulo-endothelial system. Our data suggest that hepatic ADRA2a expression is upregulated in the rodent models of liver disease we studied and aligns with literature in sepsis models, where antagonism of ADRA2a deactivates inflammatory pathways in Kupffer cells.^22^ Moreover, our data supports the contention that in the setting of high NE levels in liver disease, increased ADRA2a expression on Kupffer cells^10^^,^^13^ allows for an autocrine pro-inflammatory increase in their activation.^6^ HSCs are then activated either indirectly through inflammatory cascades, or directly via ADRA2a upregulation on HSCs. This might be expected to further aggravate intrahepatic resistance, while also promoting fibrosis progression. An important point to note, previously described in the literature as a consequence of ADRA2a antagonism, is a slight increase in plasma NE levels.^46^ This has been attributed to a block on central pre-synaptic feedback inhibition of NE release. In spite of this, we show there is reduced inflammation and improved haemodynamics, suggesting potential positive effects downstream of ADRA2a signalling.

Inflammation is a major driver of fibrosis progression in liver disease and subsequent cirrhosis decompensation and poor outcome,^47^^,^^48^ while elevated NE signalling has also been implicated in organ injury in inflammatory models.^16^ ADRA2a antagonism reduces inflammation and cytokine production and improves survival when administered after the onset of sepsis in such models.^13^ Thus, the beneficial effects of ADRA2a antagonism in liver disease are likely pleiotropic, involving both direct effects on HSCs and Kupffer cell activation, as well as indirect effects through modulation of the inflammatory hepatic milieu, as demonstrated by reduced CD68+ infiltrating cells and decreased Ccl3 and Cxcl5 chemokines in the livers of MASH animals treated with YoHCl, alongside reduced fibrogenesis. This could not only impact on fibrosis progression but clearly also on mechanisms that perpetuate portal hypertension, including inhibition of eNOS. Whether this impacts wider organ dysfunction associated with cirrhosis decompensation remains to be elucidated.

While we have focused on the role of ADRA2a on HSCs and others have described the role of ADRA2a on Kupffer cells, it is possible that ADRA2a also impacts other liver cell types. Whilst this remains a subject for future investigation, we believe it is unlikely to impact upon our understanding of the relative translational contribution of ADRA2a to fibrogenesis or portal hypertension, as investigated in this study.

A further limitation was the practicality of BRL44408 as a tool to address long-term, highly selective antagonism of ADRA2a in our MASH model, given its prohibitive costs and limited availability. However, YoHCl has a relative high selectivity for ADRA2a (with no activity on alpha-1 and beta receptors) and importantly, not only recapitulates the significant acute portal pressure reduction in BDL rats seen with BRL44408 but also reduces inflammation and fibrosis after 8 weeks treatment in our MASH model. Given YoHCl has a good safety profile and is used for other non-hepatological indications in humans, we considered this a good agent to evaluate, given opportunities for future translational studies in patients with liver disease.

Only one model was used for the portal hypertension element of this study. The BDL rat enables evaluation of a sustained inflammatory response with advancing portal hypertension, until extrahepatic organ injury ensues. This emulates the hyperdynamic circulation in patients with advanced cirrhosis, during which the autonomic system undergoes further derangement.^49^ The HFHC MASH model was chosen as the most relevant for human translation, showing fibrosis and hepatic inflammation without the introduction of a toxic cofounder, *e.g.* thioacetamide or carbon tetrachloride.^50^ The impact of such toxins on metabolic pathways, and specifically mitochondrial function, would make subsequent evaluation of potential mechanisms translatable to humans difficult. By contrast, the innate inflammatory response observed in both BDL and MASH models – likely driven in part by ADRA2a signaling and contributing to inflammatory injury as previously described^6^ – supports both their translational relevance and our choice of models.

We also acknowledge the limitation of no direct comparison between selective ADRA2a antagonism in the BDL model of portal hypertension and agents used in standard clinical care such as propranolol and carvedilol. In keeping with the principles of Replacement, Reduction and Refinement in animal research, our ethics committee advised against this. Whilst propranolol and carvedilol both lower portal pressure, both agents are well known to induce systemic hypotension and reduce liver blood flow, which we show is not the case for selective ADRA2a antagonism. Clearly, a future study of ADRA2a antagonism in patients with cirrhosis would be important to further elaborate on the results of this study.

In conclusion, our study shows that ADRA2a expression is increased in two different models of liver disease and in patients with MASH fibrosis. We demonstrate a role for ADRA2a in the pathogenesis of experimental portal hypertension and fibrogenesis and suggest this occurs through modulation of liver inflammation and HSC activation, as demonstrated by the effects of two different selective ADRA2a antagonists. These data suggest ADRA2a antagonism should be explored further as a potential target for therapeutic intervention in the management of portal hypertension and fibrosis progression.

## Abbreviations

ADRA2A, alpha 2A adrenergic receptor; ALT, alanine aminotransferase; AST, aspartate aminotransferase; BDL, bile duct ligation; CPA, collagen proportionate area; eNOS, endothelial nitric oxide synthase; HFHC, high-fat, high-cholesterol; HSCs, hepatic stellate cells; IHC, immunohistochemistry; MASLD, metabolic dysfunction-associated steatotic liver disease; MASH, metabolic dysfunction-associated steatohepatitis; MMP-2, matrix metalloproteinase-2; NE, norepinephrine; NC, normal chow; NSBB, non-selective beta-blockade; PDGFRβ, platelet-derived growth factor receptor-β; PSR, picrosirius red; ROS, reactive oxygen species; RT-qPCR, reverse transcription quantitative real-time polymerase chain reaction; SFM, serum-free medium; TIMP1, TIMP metallopeptidase inhibitor 1; YoHCl, yohimbine hydrochloride.

## Authors’ contributions

RPM conceived and designed the study, and sought funding for the project. VS, HJ, AH, MCM and ND carried out the experiments. VS, HJ, ACM, and SEA analysed the samples. RGD and MRG contributed cDNA for analysis. KLT, VS, HJ, SHD and RPM analysed and interpreted the data. KLT, VS, HJ, ACM and RPM drafted the manuscript. KLT, RJ, KR and RPM critically revised the manuscript for important intellectual content.

## Financial support

RPM received grants from Higher Education Funding UK (HEIF) and also early proof of concept funding from UCL Business (POC-12-044). KLT was supported by grants from the Danish Council for Independent Research (DFF-6110-00536) and Novo Nordic Foundation (NFF19OC0055039).

## Conflicts of interest

Manuel Romero-Gómez provided services of consulting for Abbvie, Alpha-sigma, Advanz, Apollo, Astra-Zeneca, Bausch Health, BMS, Boehringer-Ingelheim, Exo-Biologics, Gilead, Ipsen, MSD, Novo-Nordisk, Pfizer, Prosciento, Resolution Therapeutics, Roche, Rubió, Sagimet, Siemens, UCB pharma, and got research Grants from Gilead, Intercept, Siemens, Theratechnologies, Novo-Nordisk, Echosens. Krista Rombouts ownes shares or received stock options and receives consultancies in Engitix Therapeutics Ltd. Rajiv Jalan is founder of Yaqrit Limited, a spin out company from University College London. He also is co-founder of Hepyx Limited and Cyberliver Limited. Rajeshwar Prosad Mookerjee is a co-founder of Yaqrit Limited, and also has scientific collabaorations with Cyberliver Limited and Hepyx Limited. He is co-inventor of patents filed by UCL Business around ADRA2a, now licensed to Hepyx Limited. All other authors have no conflicts to declare.

Please refer to the accompanying ICMJE disclosure forms for further details.
