# Supplementary material for: Alpha 2A adrenergic receptor antagonism reduces fibrosis, inflammation and portal hypertension in models of chronic liver disease
Source: JHEP Rep. 2026 Jun 9;8(9):101922. doi: 10.1016/j.jhepr.2026.101922 (PMC13453047; doi:10.1016/j.jhepr.2026.101922)
Supplement: Supplementary file 1 [file mmc1.pdf]

Alpha 2A adrenergic receptor antagonism reduces fibrosis, inflammation and portal hypertension in models of chronic liver disease

Karen Louise Thomsen, Vikram Sharma, Helen Jones, Anne Catrine Daugaard Mikkelsen, Nathan Davies, Abeba Habtesion, Sara Ellegaard Paaske, Jane Macnaughtan, Maria Castanho Martins, Rocio Gallego-Durán, Manuel Romero-Gómez, Stephen Hamilton-Dutoit, Krista Rombouts, Rajiv Jalan, Rajeshwar Prosad Mookerjee

Table of contents

Supplementary materials and methods……………………………………………………2

Table S1……………………………………………………………………………………..14

Supplementary references…………………………………………………………….....15

SUPPLEMENTAL MATERIALS AND METHODS

Animals

Male Sprague Dawley rats (body weight 220-300 g; Charles River, UK) were housed in a temperature and light controlled (12 hours light/dark cycle) facility at University College London. All animals were allowed to acclimatise for a week followed by randomization. All procedures were performed in accordance with UK Home Office Animals (Scientific Procedures) Act 1986 Amendment Regulations 2012, following approval from the local animal welfare and ethical review board. The animal experiments are reported in accordance with the ARRIVE guidelines (1).

BDL animal model: Rats underwent bile duct ligation (BDL) as described previously (2, 3), whilst sham-operated rats had a laparotomy without BDL (4). Four weeks after BDL or sham operation, rats were allocated either: (i) Sham-operated (control) rats administered subcutaneous (sc) saline BD, (ii) BDL rats administered sc saline BD or (iii) BDL rats receiving sc injection of the selective ADRA2a antagonist BRL44408 (Sigma-Aldrich Company Ltd., Dorset, UK) (10 mg/kg) BD within 24 hours prior to haemodynamic measurements. Also, sham-operated rats administered sc BRL44408 BD were included for haemodynamic measurements. Similarly, a cohort of BDL rats were also administered Yohimbine hydrochloride (YoHCl) (Sigma-Aldrich) (0.4mg/kg) for 2 doses, within 24 hours of haemodynamic assessment.

For all groups, animals were anaesthetized (induction 5% Isoflurane in oxygen, maintenance 1% in room air) and a cannula placed in the left carotid artery for continuous mean arterial pressure (MAP) monitoring (Biopac US). Following a laparotomy, a sub-group of animals underwent portal and hepatic arterial blood flow measurement (Transonic US). The portal pressure was

measured at the end of the procedure by inserting a 25G needle attached to a pressure transducer directly into the portal vein and values recorded after a period of stabilization, as we have shown previously (5).

Metabolic dysfunction-associated steatohepatitis (MASH) animal model: Twenty-four rats were fed normal chow (NC) (RM1; Special Diet Services, Witham, UK) or a high-fat, high-cholesterol (HFHC) diet (D09052204Y; Research Diets, USA) for 16 weeks as described previously (6, 7). The standard diet was composed of the following energy sources: 67 g carbohydrates (70 kcal/100 kcal), 4 g fat (10 kcal/100 kcal), and 19 g protein (20 kcal/100 kcal) per 100 g diet. The HFHC diet contained 19 g carbohydrates (15 kcal/100 kcal), 39 g fat (65 kcal/100 kcal), 2 g cholesterol, and 27 g protein (20 kcal/100 kcal) per 100 g diet. The fat was mainly from cocoa butter. The diets were provided *ad libitum* and the daily leftovers were weighed to determine the caloric intake. For the final 8 weeks of the study, HFHC fed rats were randomly allocated to receive YoHCl (Sigma-Aldrich) in their drinking water or control. Fluid was adjusted to achieve an average dose of 0.4 mg/kg/day. The animal groups were: (i) NC group: NC for 16 weeks (n=9), (ii) HFHC group: HFHC diet for 16 weeks (n=6) and (iii) HFHC+YoHCl group: HFHC diet and YoHCl for a final 8 weeks of study (n=9).

All rats from both experimental protocols were exsanguinated under terminal anaesthesia; blood was withdrawn into heparin or EDTA vacutainers (BD Biosciences, Oxford, UK), centrifuged (3500 rpm, 10 min, 4°C), and plasma stored at -80°C until further analyses. Liver was weighed and tissue samples were snap frozen in liquid nitrogen and stored at −80°C or fixed in 10% neutral buffered formalin. All of the assays used in this study had been validated for use in rats.

Hepatic Stellate Cell (HSCs)

Cell isolation and culture

Primary human (h)HSCs were isolated according to Mederacke et al (8) with modifications for human liver (9) (REC reference 21/WA/0388). HSCs were isolated from normal human liver (i.e. without inflammation and fibrosis) (10), obtained after liver wedge section surgery in patients with primary liver cancer or liver metastasis from extrahepatic cancers (9). Briefly, 10 g of total human liver tissue was digested with 0.01% Collagenase, 0.05% Pronase and 0.001% DNase I without performing perfusion. The homogenate was filtered through a 100m cell strainer (BD Biosciences) and the flow-through was centrifuged at 50g for 2 min at 4°C. After washing the supernatant, gradient centrifugation was performed at 1400g for 17 min at 4°C using an 11.5% Optiprep gradient (Sigma-Aldrich). Finally, the interface was collected and washed. Purity of hHSC was established by detection of CD140b (PDGFRbeta), CD29 (Integrin beta 1) and Cytoglobin B (CYGB).

Cells were cultured in complete medium (CM) containing Iscove's Modified DMEM (IMDM, Thermo Fisher Scientific, Watlham, USA), supplemented with 20% foetal bovine serum (FBS, Thermo Fisher Scientific), 2 mM Glutamine, 1X non-essential amino acids, 1.0 mM sodium pyruvate, 1X antibiotic-antimycotic (Life Technologies, Carlsbard, USA), and maintained under standard conditions in a humidified incubator under 5% CO_2 in air at 37°C. Medium was refreshed twice a week and cells were passaged when subconfluent with 1X Trypsin (Gibco, Thermo Fisher Scientific) (9). Rat HSCs cells were a kind gift from Jonathan Fallowfield (University of Edinburgh). Rat HSCs were cultured in DMEM (high glucose, with sodium pyruvate & Glutamax) with 10% FBS (Life Technologies).

Treatments

All treatments with agonist or antagonist were performed in serum free medium (SFM). Human HSCs were seeded at 300,000 cells per well in CM in 6-well plates. After 24 hours CM was replaced with SFM for a further 24 hours to induce quiescence. Cells were subsequently treated with SFM, 1% FBS-CM (CM with FBS reduced to 1%), CM, or SFM + 5uM guanfacine hydrochloride (CAS: 29110-48-3; Sigma-Aldrich) for 24 hours.

Gel contraction

Human and rat HSCs were cultured within 3D collagen gels as previously described (11). Twenty four well cell culture plates were pre-coated with sterile 2% BSA (Sigma-Aldrich) in phosphate buffered saline (PBS). Ice-cold neutral collagen solution was made by mixing 1 part 0.2M HEPES pH 8.0 (Life Technologies), 4 parts >2mg/ml rat tail collagen type I (First Link (UK) Ltd., Wolverhampton, UK), and 5 parts SFM. Equal volumes of the neutral collagen solution and HSCs resuspended at 80.000 cells/ml in SFM or SFM with 5uM BRL44408 (CAS: 118343-19-4; Tocris Bioscience, Abingdon, UK) were mixed. One ml was added to each well and allowed to polymerise for ≥2 hours at 37°C 5% CO_2. After polymerisation, 1ml of SFM, SFM with 5uM guanfacine, SFM with 50uM guanfacine, or SFM with 5uM guanfacine and 5uM BRL44408 was added causing detachment of the gel from the plate. After 6 hours the contracted gels were imaged using a Fluorchem M imager (ProteinSimple) with white light transillumination and 607/36 nm filter. Gel areas were measured using ImageJ v1.49 software (12).

RT-qPCR of primary human HSCs

In hHSCs, mRNA expression of ADRA2A and PDGFR-β were determined by RT-qPCR. RNA was extracted from cell pellets of 14 donors using the Rneasy Mini kit (Qiagen Ltd., Manchester, UK). RNA concentration was measured using the Qubit RNA Broad Range assay (Invitrogen, UK). A total amount of 800ng of each sample were reverse transcribed using the High-Capacity cDNA Reverse Transcription Kit (Thermo Fisher Scientific) according to manufacturer's instructions. qPCR was performed using TaqMan Fast Advanced Master Mix (Thermo Fisher) with Assays-on-Demand primers (Thermo Fisher) on a QuantStudio 7 Pro (Applied Biosystems). Data was analysed using the delta Ct method with GAPDH as endogenous control.

Metabolic dysfunction-associated steatotic liver disease (MASLD) patients

Fifteen MASLD patients were recruited from the Hepatology Unit of Virgen del Rocío University Hospital, Spain. Patients were ≥18 years of age and MASLD was diagnosed based on the following inclusion criteria: a) ultrasonographic evidence of hepatic steatosis; b) elevated liver enzymes; c) metabolic syndrome following ATPIII criteria (13). Exclusion criteria were as follows: significant alcohol intake (≥30 g/day in men and ≥20 g/day in women), recreational drugs abuse, and evidence of viral or autoimmune hepatitis, HIV, drug-induced fatty liver or other metabolic liver diseases. Study procedures agreed with the ethical standards of the responsible committee on human experimentation and were approved by the human research ethics committee (C.I. 0359-N-15). Informed consent was ontained from all participants.

Percutaneous liver biopsies were obtained after an overnight fast and performed under local anaesthesia and ultrasound guidance using a tru-cut needle. The biopsy samples were divided in two and immediately snap-frozen and stored at -80ºC or formalin-fixed overnight and paraffin-embedded. The liver specimens were stained with haematoxylin-eosin, reticulin and Masson´s

trichrome. An experienced pathologist unaware of clinical data assessed the samples to determine the Non-alcoholic fatty liver disease (NAFLD) Activity Score (NAS) and fibrosis stage by Kleiner Score (14).

Messenger RNA expression of ADRA2a was evaluated using RT-qPCR. Total RNA was isolated from the snap-frozen liver specimens using a mirVana Isolation kit (Life Technologies) following manufacturer´s instructions. RNA Integrity number (RIN) was measured with an Agilent 2100 Bioanalyzer System, and RNA concentration was evaluated using a Nanodrop One (Thermo Fisher Scientific). Reverse transcription was performed on 500 ng per patient sample using the PrimeScript RT Master Mix (Takara, Shiga, Japan). qPCR was performed as described for the animal studies.

Blood analyses

Monocyte Phagocytic Studies

Whole blood preparation: 2 ml of blood was collected from the portal vein. 40 ml of RBC lysis buffer was added to each 2 ml sample, vortexed and incubated for 15 min at room temperature. Following centrifugation at 450g for 5 min, the supernatant was discarded, and the pellet re-suspended with 1 ml of complete culture media (RPMI (Gibco), 10% FBS, 200mM L-Glutamine). The cell number was assessed using the nucleoCounter method and adjusted to $1x10^7$ cell/ml.

The cells were centrifuged at 500g for 5 min at 4ºC and the supernatant discarded. 200 µl media containing latex beads (L2778, Sigma) were added to the pellet and incubated at 37°C in the dark for 20 min. 5 ml of ice-cold PBS was then added and centrifuged at 800g for 5 min at 4ºC. The pellet was then washed with 5 ml of cold PBS and centrifuged. Cells were incubated with anti-

CD32 antibody for 10 min at 4°C followed by incubation with PE-conjugated anti-CD11b (MCA275A647 (Clone OX-42), Abd Serotec) and A647-conjugated anti-CD43 (202810 (Clone W3/13), Biolegend) or isotype control for 30 min at 4°C in the dark. The cells were then washed with 1ml of FACS buffer, centrifuged and re-suspended in 100 µl FACS buffer solution. Samples were kept at 4°C and analysed immediately using a FACS LSR II Flow Cytometer (Becton Dickinson). Data was analysed using FlowJo software.

Liver tissue analyses

Collagen proportionate area (CPA) measurement

CPA was determined on NanoZoomer images of PSR stained whole sections of liver. Images were captured at 20x magnification and analysis was performed on 5x magnification TIFF images extracted from the NDPI files using the NDPI-split tool (15). All subsequent digital image analysis was performed using ImageJ2 (16, 17). A region of interest (ROI) covering the entire liver section excluding only cutting artifacts and lumen of large vessels was selected for analysis. A custom macro script was written to measure the total area of the ROI, threshold the image to segregate only the red-stained collagen and measure the area of the thresholded portion of the ROI. The script was run in batch mode for all the images analysed. CPA was calculated as the thresholded ROI area as a percentage of the total ROI area.

Western blot analysis

In BDL animals, protein expression of ADRA2a, caveolin-1, eNOS and p-eNOS was measured using WB analysis as described previously (11). Proteins were isolated from fresh-frozen liver tissues, which were homogenized in lysis buffer containing 50 mM Tris-HCl (pH 7.6) with a

protease inhibitor cocktail (Sigma-Aldrich), protein methyl sulphonyl fluride (PMSF) and ethylene diamine tetra acetic acid (EDTA) using a tissue homogenizer. The lysates were centrifuged for 60 min at 10,000 rpm and the supernatants measured for protein concentration by the Bradford method. Extracts containing equal amounts of protein was denatured and separated on 4-12% NuPAGE Bis-Tris Gels and blotted on to PVDF membranes (Invitrogen). The membranes were then incubated with different primary antibodies, and specific protein bands detected using HRP-conjugated secondary antibodies with an enhanced ECL detection kit, rabbit anti-α tubulin (1:1000; Upstate, Biotechnology, N.Y.) serving as a loading control.

RNA isolation and RT-qPCR

In MASH animals, mRNA expression of ADRA2A and TIMP metallopeptidase inhibitor 1 (TIMP1) was determined using RT-qPCR. Frozen liver was homogenized in TRI reagent (Sigma-Aldrich) using 7mm steel beads with a TissueLyser LT (Qiagen Ltd.). After extraction with chloroform total RNA was isolated using a RNeasy mini kit (Qiagen Ltd.). RNA concentration was measured using a nanodrop (Labtech International Ltd., Uckfield, UK). Reverse transcription was performed on 1ug RNA per sample with combined polyT and random hexamer primers using a Quantitect reverse transcription kit (Qiagen Ltd.). A no-RT control reaction omitting the reverse transcriptase enzyme was also performed. qPCR was performed using hydrolysis probe assays (Integrated DNA Technologies, BVBA., Leuven, Belgium) and Rotor-gene probe PCR mix (Qiagen Ltd.) on a Rotor-gene 6000 (Qiagen Ltd.). Data was analysed by calculating relative quantity (RQ; $2^{\Delta ct}$) using the average of the NC group as the calibrator. RQ was normalised using a normalisation factor generated from the geometric mean of RQ of 3 reference genes (Tbp, Ppia, Hmbs) (18).

Hepatic MMP-2 protein expression

In MASH animals, protein expression of active MMP-2 was measured using gel zymography. Frozen liver was homogenised in RIPA buffer (Pierce Biotennchnology Inc., Rockford IL, USA) with complete ultra mini EDTA-free protease inhibitors and phoSTOP phosphatase inhibitors (Roche Diagnostics Ltd.) using 7mm steel beads with a TissueLyser LT (Qiagen Ltd.). Protein concentration was measured using a BCA assay kit (Pierce Biotechnology Inc.). Equal amounts of protein were separated on 10% Novex Tris-Glycine gels containing 0.1% gelatine under non-reduced denaturing conditions using tris-glycine-SDS running buffer (Life Technologies). The molecular weight markers were Novex Sharp prestained standard (Life Technologies). Following electrophoresis, the gels were incubated in zymogram renaturing buffer (Life Technologies) and then developed overnight at 37 °C in zymogram developing buffer (Life Technologies). The developed gels were stained overnight in an excess of GelCode Blue Safe stain (Sigma-Aldrich) and imaged using a UVP VioSpectrum imaging system (UVP Ltd, Cambridge, UK). Densitometry was measured using ImageJ2 (16, 17).

Hepatic protein expression of chemokines

Hepatic protein expression of the selected chemokines C-C motif ligand 3 (Ccl3), C-X3-C motif ligand 1 (Cx3cl1) = fractalkine and C-X-C motif chemokine 5 (Cxcl5) was then assessed by ELISA. Frozen liver was homogenised in lysis buffer containing sodium deoxycholate (RABLYSIS1; Sigma-Aldrich) with protease phosphatase inhibitors as above. Protein concentration was measured by BCA. Sandwich ELISAs were used to measure Ccl3 (20ug protein per well; Cloud-Clone Corp, Katy, TX, USA), Cx3Cl1 (20ug protein per well; RAB0115; Sigma-

Aldrich), and Cxcl5 (100ug protein per well; ERCXCL5CL; Life Technologies) according to the manufacturers' instructions in a 96 well plate (as above). .

Hepatic CD68 protein expression

In MASH animals, 3-5mm liver sections were dewaxed in xylene and rehydrated through graded alcohols prior to immunohistochemical (IHC) detection of CD68. Heat-induced epitope retrieval was performed by boiling for 20mins in 10mM sodium citrate pH 6 in a microwave. Blocking steps consisted of avidin/biotin blocking (Vector Laboratories Ltd., Peterborough, UK), general protein blocking (2.5% normal horse serum; Vector Laboratories Ltd.), and peroxidase blocking (3% H_2O_2). Blocked sections were incubated overnight at 4°C in biotinylated mouse anti-CD68 (1:100 dilution). Secondary detection was performed using HRP-conjugated ABC reagent (VectaStain Elite; Vector Laboratories Ltd.) and DAB substrate (Vector Laboratories Ltd.). Stained sections were counterstained using Gills haematoxylin (Sigma-Aldrich) and mounted in DPX. Slides were digitised and quantified as above (15). All subsequent digital image analysis was performed using ImageJ2 (16, 17). A ROI covering the entire liver section excluding only cutting artefacts and lumen of large vessels was selected for analysis. A custom macro script was written to measure the total area of the ROI, perform colour deconvolution to separate the DAB stain from the haemotoxylin counter-stain, threshold the image to segregate only the DAB-stained regions, and measure the area of the thresholded portion of the ROI. The script was run in batch mode for all the images analysed. IHC-stained area was calculated for the thresholded ROI area as a percentage of the total ROI area.

Kupffer cell phagocytic function and ROS production

Isolation of non-parenchymal cell fraction from rodent liver tissue:

The liver was perfused with 5 ml of ice-cold PBS and homogenized in Hanks balanced salt solution (HBSS) (Ca^{2+} and Mg^{2+}) with collagenase (0.01%) and DNase I (0.001%) (Gibco). The homogenate was transferred to a 50 ml Falcon tube and incubated at 37°C for 20 min prior to filtration through a 100 μm cell strainer. This was then centrifuged at 50g for 2 min at 4°C and the supernatant then centrifuged at 450g for 10 min at 4°C. The supernatant was discarded, and the pellet re-suspended in HBSS (no Ca^{2+} and Mg^{2+}), DNase I 0.01%, bovine serum albumin (0.25%) and centrifuged at 450g for 10 min at 4°C. The pellet was then re-suspended in 3.9 ml of RPMI 1640 and mixed gently with 2.1 ml of RPMI (Thermo Fisher Scientific) and Optiprep 22% (Stemcell Technologies). RPMI was then layered on top followed by a 25-minute centrifugation step at 1500g without brake at 4°C. The non-parenchymal cells were isolated from the interface, re-suspended in an equivalent volume of HBSS (free Ca^{2+} and Mg^{2+}), DNase I 0.01%, bovine serum albumin (0.25%) and centrifuged at 800g at 4°C for 10 min. The pellet was re-suspended in 5 ml of Red Cell Lysis Buffer (BioLegend) and incubated for 5 min at 4°C with occasional shaking. The reaction was then stopped by addition of 10 ml of PBS. This was then centrifuged at 800g for 10 min at 4ºC, the supernatant discarded, and the cells re-suspended in 3ml of culture media. The cells were counted and adjusted to a concentration of 10^7 cells/ml. $1x10^6$ cells were used in all subsequent assays.

Kupffer cell population studies:

2 μl anti-CD32 antibody (550270 (Clone D34-485), Becton Dickinson) was added to 10^6 cells (non-parenchymal cell fraction) and incubated for 5 min at 4°C. The cells were then co-incubated with anti-CD163 antibody (MCA342A647 (Clone ED2), AbD Serotec) and anti-CD68 antibody

(MCA341B, (Clone ED1), Abd Serotec) or isotype controls for 30 min at 4°C in the dark. The cells were washed with 1 ml of FACS buffer (42020, Cell staining buffer, BioLegend), centrifuged and re-suspended in 100 µl FACS buffer solution and analysed immediately on a Becton Dickinson LSR II flow cytometer. Data was analysed using FlowJo software.

Kupffer cell ROS studies:

20 µg/ml of E.coli endotoxin was added to 1x 10^6 non-parenchymal cells sample and incubated for 30 min at 37°C. ROS inducer at a final concentration of 200-500uM was used as a positive control. The samples were then centrifuged at 500g for 5 min and the supernatant discarded. The cells were re-suspended in 5 ml of wash buffer, centrifuged at 500g for 5 min and the supernatant removed. The cells were re-suspended in 500 µl of ROS detection solution and incubated for 30 min at 37°C in the dark. Following centrifugation, the cells were re-suspended in 100 µl of FACS buffer, and incubated with anti-CD32 antibody (550270 (Clone D34-485) for 10 min at 4°C. Cells were then incubated with anti-CD163 antibody or isotype control for 30 min at 4°C in the dark. The cells were then washed with 1 ml of FACS buffer, centrifuged and re-suspended in 100 µl FACS buffer solution. samples were kept at 4°C and analysed immediately using a FACS LSR II Flow Cytometer (Becton Dickinson). Data was analysed using FlowJo software.

Table S1.
Clinical and biochemical characteristics and histological scores

	F0 fibrosis	F1-3 fibrosis
Age (years)	42 ± 11	56 ± 12
Sex (m/f)	4/3	5/3
BMI	28.6 ± 7.1	32.7 ± 1.9
ALT (U/L)	33 ± 15	54 ± 30
AST (U/L)	28 ± 10	47 ± 18
HOMA	2.7 ± 1.6	4.0 ± 3.0
NAS score	1.5 ± 0.9	3.4 ± 1.1
Fibrosis score	0.0 ± 0.0	2.1 ± 0.9

Age, sex, body mass index (BMI) and plasma concentrations of alanine aminotransferase (ALT) and aspartate aminotransferase (AST), homeostatic model assessment (HOMA) and histological scores of liver tissue (non-alcoholic fatty liver disease (NAFLD) activity score (NAS) and fibrosis) in patients with metabolic dysfunction-associated steatotic liver disease and F0 (n=7) and F1-3 (n=8) fibrosis.

Supplementary references

1. Percie du Sert N, Hurst V, Ahluwalia A, Alam S, Avey MT, Baker M, et al. The ARRIVE guidelines 2.0: Updated guidelines for reporting animal research. PLoS Biol. 2020;18(7):e3000410.
2. Kountouras J, Billing BH, Scheuer PJ. Prolonged bile duct obstruction: a new experimental model for cirrhosis in the rat. Br J Exp Pathol. 1984;65(3):305-11.
3. Poo JL, Estanes A, Pedraza-Chaverri J, Cruz C, Perez C, Huberman A, et al. [Chronology of portal hypertension, decreased sodium excretion, and activation of the renin-angiotensin system in experimental biliary cirrhosis]. Rev Invest Clin. 1997;49(1):15-23.
4. Harry D, Anand R, Holt S, Davies S, Marley R, Fernando B, et al. Increased sensitivity to endotoxemia in the bile duct-ligated cirrhotic Rat. Hepatology. 1999;30(5):1198-205.
5. Mookerjee RP, Mehta G, Balasubramaniyan V, Mohamed Fel Z, Davies N, Sharma V, et al. Hepatic dimethylarginine-dimethylaminohydrolase1 is reduced in cirrhosis and is a target for therapy in portal hypertension. J Hepatol. 2015;62(2):325-31.
6. Thomsen KL, Gronbaek H, Glavind E, Hebbard L, Jessen N, Clouston A, et al. Experimental nonalcoholic steatohepatitis compromises ureagenesis, an essential hepatic metabolic function. Am J Physiol Gastrointest Liver Physiol. 2014;307(3):G295-301.
7. De Chiara F, Thomsen KL, Habtesion A, Jones H, Davies N, Gracia-Sancho J, et al. Ammonia Scavenging Prevents Progression of Fibrosis in Experimental Nonalcoholic Fatty Liver Disease. Hepatology. 2020;71(3):874-92.
8. Mederacke I, Dapito DH, Affo S, Uchinami H, Schwabe RF. High-yield and high-purity isolation of hepatic stellate cells from normal and fibrotic mouse livers. Nat Protoc. 2015;10(2):305-15.
9. Rombouts K, Carloni V. Determination and Characterization of Tetraspanin-Associated Phosphoinositide-4 Kinases in Primary and Neoplastic Liver Cells. Methods Mol Biol. 2016;1376:203-12.
10. Caon E, Martins M, Hodgetts H, Blanken L, Vilia MG, Levi A, et al. Exploring the impact of the PNPLA3 I148M variant on primary human hepatic stellate cells using 3D extracellular matrix models. J Hepatol. 2024;80(6):941-56.
11. Jalan R, De Chiara F, Balasubramaniyan V, Andreola F, Khetan V, Malago M, et al. Ammonia produces pathological changes in human hepatic stellate cells and is a target for therapy of portal hypertension. J Hepatol. 2016;64(4):823-33.
12. Schneider CA, Rasband WS, Eliceiri KW. NIH Image to ImageJ: 25 years of image analysis. Nature Methods. 2012;9(7):671-5.
13. Expert Panel on Detection E, Treatment of High Blood Cholesterol in A. Executive Summary of The Third Report of The National Cholesterol Education Program (NCEP) Expert Panel on Detection, Evaluation, And Treatment of High Blood Cholesterol In Adults (Adult Treatment Panel III). JAMA. 2001;285(19):2486-97.
14. Kleiner DE, Brunt EM, Van Natta M, Behling C, Contos MJ, Cummings OW, et al. Design and validation of a histological scoring system for nonalcoholic fatty liver disease. Hepatology. 2005;41(6):1313-21.
15. Deroulers C, Ameisen D, Badoual M, Gerin C, Granier A, Lartaud M. Analyzing huge pathology images with open source software. Diagn Pathol. 2013;8:92.
16. Schindelin J, Arganda-Carreras I, Frise E, Kaynig V, Longair M, Pietzsch T, et al. Fiji: an open-source platform for biological-image analysis. Nat Methods. 2012;9(7):676-82.
17. Rueden CT, Schindelin J, Hiner MC, DeZonia BE, Walter AE, Arena ET, et al. ImageJ2: ImageJ for the next generation of scientific image data. BMC Bioinformatics. 2017;18(1):529.
18. Hellemans J, Mortier G, De Paepe A, Speleman F, Vandesompele J. qBase relative quantification framework and software for management and automated analysis of real-time quantitative PCR data. Genome Biol. 2007;8(2):R19.
