# Supplementary material for: Alpha 2A adrenergic receptor antagonism reduces fibrosis, inflammation and portal hypertension in models of chronic liver disease
Source: JHEP Rep. 2026 Jun 9;8(9):101922. doi: 10.1016/j.jhepr.2026.101922 (PMC13453047; doi:10.1016/j.jhepr.2026.101922)
Supplement: Supplementary file 2 [file mmc2.pdf]

JHEP|Reports

JHEP Reports

CTAT methods

Tables for a "Complete, Transparent, Accurate and Timely account" (CTAT) are now mandatory for all revised submissions. The aim is to enhance the reproducibility of methods.

- Only include the parts relevant to your study
- Refer to the CTAT in the main text as 'Supplementary CTAT Table'
- Do not add subheadings
- Add as many rows as needed to include all information
- Only include one item per row

If the CTAT form is not relevant to your study, please outline the reasons why:

1.1 Antibodies

Name	Citation	Supplier	Cat no.	Clone no.
All mentioned in Suppl. Materials and Methods				

1.2 Cell lines

Name	Citation	Supplier	Cat no.	Passage no.	Authentication test method
N/A					

1.3 Organisms

Name	Citation	Supplier	Strain	Sex	Age	Overall n number
Sprague Dawley rats		Charles River, UK		Male	12 weeks	65
Primary Human Hepatic Stellate Cells		Human healthy liver tissue, Royal Free Hospital, London, UK				

1.4 Sequence based reagents

Name	Sequence	Supplier
N/A		

1.5 Biological samples

Description	Source	Identifier
Liver biopsies	MASLD patients, the Virgen del Rocío University Hospital, Spain	

1.6 Deposited data

Name of repository	Identifier	Link
N/A		

1.7 Software

Software name	Manufacturer	Version
N/A		

1.8 Other (*e.g.* drugs, proteins, vectors etc.)

BRL44408	Sigma-Aldrich, Dorset, UK	
Yohimbine hydrochloride	Sigma-Aldrich, Dorset, UK	

1.9 Please provide the details of the corresponding methods author for the manuscript:

Rajeshwar Prosad Mookerjee

2.0 Please confirm for randomised controlled trials all versions of the clinical protocol are included in the submission. These will be published online as supplementary information.

N/A
