# Supplementary material for: Alpha 2A adrenergic receptor antagonism reduces fibrosis, inflammation and portal hypertension in models of chronic liver disease
Source: JHEP Rep. 2026 Jun 9;8(9):101922. doi: 10.1016/j.jhepr.2026.101922 (PMC13453047; doi:10.1016/j.jhepr.2026.101922)
Supplement: Supplementary file 4 [file mmc4.pdf]

JHEP Reports

Alpha 2A adrenergic receptor antagonism reduces fibrosis, inflammation and portal hypertension in models of chronic liver disease

Authors

Karen Louise Thomsen, Vikram Sharma, Helen Jones, …, Krista Rombouts, Rajiv Jalan, Rajeshwar Prosad Mookerjee

Correspondence

r.mookerjee@ucl.ac.uk (R.P. Mookerjee).

Graphical abstract

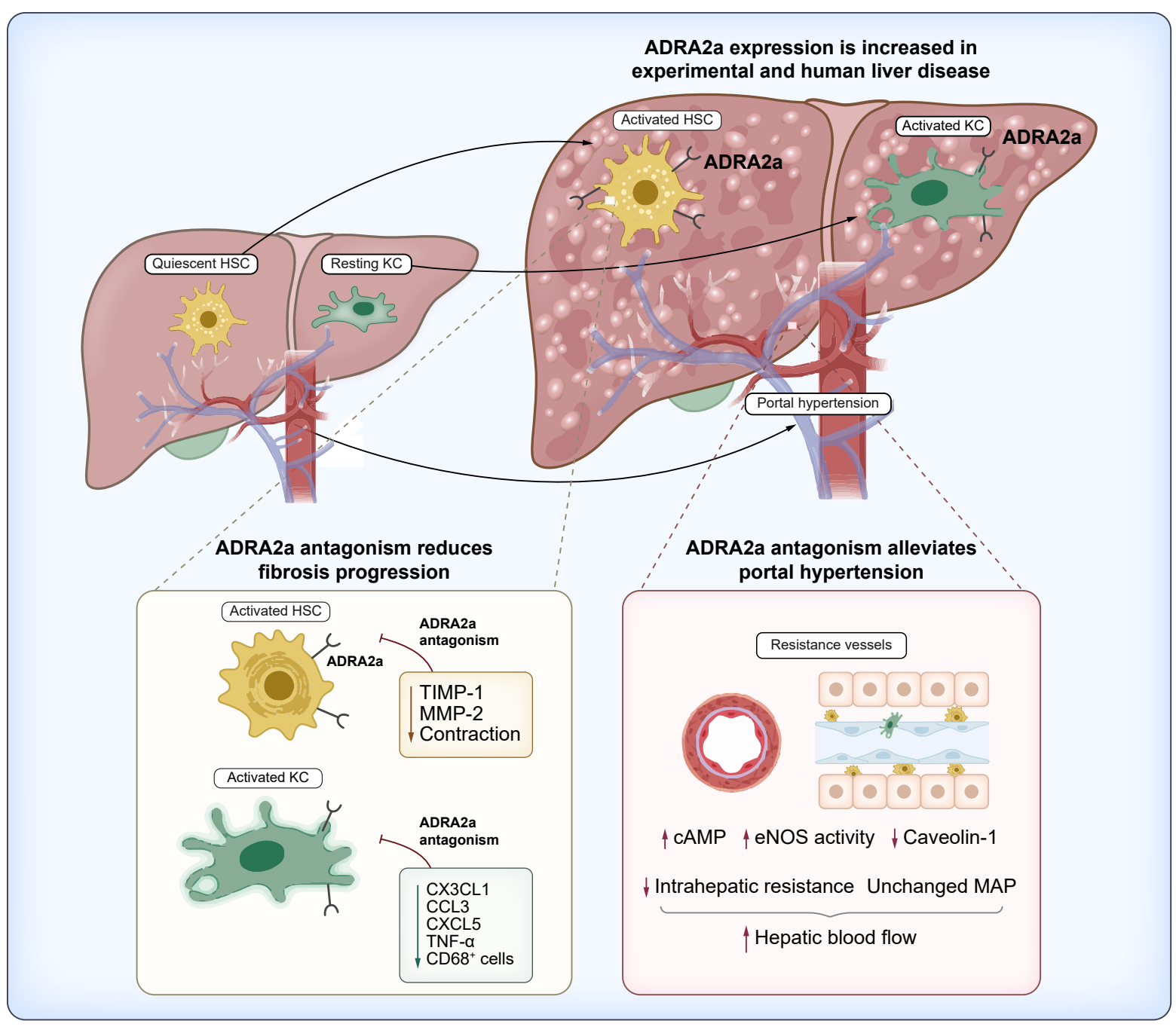

Highlights:

- Hepatic ADRA2a expression increases in liver disease.
- Antagonists of ADRA2a decrease stellate cell activation in culture.
- ADRA2a antagonism reduces portal hypertension and fibrosis in liver disease models.
- ADRA2a antagonism may be a potential therapeutic target in evolving liver disease.

Impact and implications:

Managing fibrosis progression and portal hypertension remains a major challenge in liver disease, with fewer than 60% of patients responding to current non-selective beta-blocker therapy, despite clear evidence of increased sympathetic activation as liver disease advances. We show that the alpha-2A adrenergic receptor (ADRA2A) may represent an important pathway in hepatic stellate cell activation and may also influence additional mechanisms that regulate elevated portal pressure. These findings provide an alternative approach to beta-blockade for portal hypertension, which is limited by reductions in cardiac output and liver blood flow that can be problematic in patients with advanced disease. Data from two different rodent models support consideration of a translational clinical study of ADRA2A antagonism in portal hypertension and further investigation into the mechanisms by which ADRA2A antagonism affects metabolic dysfunction-associated steatotic liver disease.

https://doi.org/10.1016/j.jhepr.2026.101922

© 2026 The Authors. Published by Elsevier B.V. on behalf of European Association for the Study of the Liver (EASL). This is an open access article under the CC BY license (http://creativecommons.org/licenses/by/4.0/). JHEP Reports, 2026, 8, 1–12

JHEP|Reports

Alpha 2A adrenergic receptor antagonism reduces fibrosis, inflammation and portal hypertension in models of chronic liver disease

Karen Louise Thomsen[1,2,†], **Vikram Sharma**[1,†], **Helen Jones**[1,†], Anne Catrine Daugaard Mikkelsen[2], Nathan Davies[1], Abeba Habtesion[1], Sara Ellegaard Paaske[2], Jane Macnaughtan[1], Maria Castanho Martins[1], Rocio Gallego-Durán[1,3], Manuel Romero-Gómez[3], Stephen Hamilton-Dutoit[4], Krista Rombouts[1], Rajiv Jalan[1], Rajeshwar Prosad Mookerjee[1,2,*]

JHEP Reports **2026**. vol. 8 | 1–12

Check for updates

Background & Aims: Sympathetic nervous system over-activity is associated with liver disease progression and development of portal hypertension, influencing systemic inflammation. This study examined the role of the sympathetic alpha-2A adrenergic receptor (ADRA2a) and its antagonism in hepatic stellate cell (HSC) activation, a key event in fibrosis progression, in experimental metabolic dysfunction-associated steatohepatitis (MASH) and in portal pressure elevation in a cirrhotic-rat model.

Methods: An established bile duct ligation (BDL)-induced cirrhosis model (n = 14) was used to assess ADRA2a's role in portal hypertension through acute treatment with two ADRA2A antagonists (BRL44408 and yohimbine). Human (h)HSCs were incubated with either an ADRA2a agonist (guanfacine) or antagonist (BRL44408), to determine whether ADRA2a modulation altered HSC activation. We also investigated ADRA2a expression in patients with MASH fibrosis (n = 15) and conducted a longer-term yohimbine treatment study in a diet-induced MASH rodent model (n = 24).

Results: BRL44408 reduced portal pressure in BDL rats (12 ± 3 *vs.* 18 ± 4 mmHg; p <0.0001) while preserving mean arterial pressure (102 ± 16 *vs.* 93 ± 13 mmHg, p = 0.13). This was associated with (i) restored eNOS phosphorylation towards control levels and reduced caveolin-1 expression (p <0.05), and (ii) reduced hepatic inflammation and Kuppfer cell activation (p <0.001). Moreover, guanfacine stimulation of hHSCs increased their contractility, which was attenuated by BRL44408 (p <0.001). *ADRA2A* mRNA expression was increased in both patients with MASH fibrosis, and MASH rats. In MASH rats, yohimbine treatment reduced fibrosis, as measured by collagen proportional area (p <0.01).

Conclusions: ADRA2A expression is increased in two experimental models of liver disease and in patients with MASH fibrosis, and it appears to contribute to the pathogenesis of portal hypertension and fibrogenesis. These findings suggest that ADRA2A antagonism may represent a potential therapeutic strategy for treating portal hypertension and fibrosis progression.

© 2026 The Authors. Published by Elsevier B.V. on behalf of European Association for the Study of the Liver (EASL). This is an open access article under the CC BY license (http://creativecommons.org/licenses/by/4.0/).

Introduction

The pathophysiology of portal hypertension is well described,[1] combining hepatic architectural changes, hepatic stellate cell (HSC) activation and endothelial dysfunction. Sympathetic nervous system overactivity is associated with liver disease progression and the development of portal hypertension; however, the limited response to portal pressure reduction with non-selective beta-blockers (NSBBs) (40–50%)[2] highlights the need to consider other mechanistic targets.

Of interest, both steatosis and metabolic dysfunction-associated steatohepatitis (MASH) are associated with increasing portal pressure in animal models and patients, even in the absence of fibrosis progression.[3] As MASH progresses, chronic inflammation and hepatocyte injury ultimately lead to fibrogenesis, associated with long-term morbidity and mortality.[4] The main cell type responsible for extracellular matrix deposition is the HSC, which is also a key pathophysiological driver in the development of portal hypertension.[5] Current licensed treatments for MASH, until recently, have largely been limited to modification of risk factors such as obesity and diabetes melitus.

The autonomic nervous system influences systemic inflammation.[6] Levels of norepinephrine (NE) are elevated in acute decompensation of cirrhosis and correlate with increased intrahepatic resistance, changes in organ blood flow and severity of inflammation.[7,8] NE metabolites have recently been linked with progression of acute cirrhosis decompensation.[9] Moreover, animal models of sepsis suggest that NE participates in the development of hepatocellular dysfunction[6,10] and that the gut and innate immune system are major

* Corresponding author. Address: UCL Institute for Liver and Digestive Health, University College London, Royal Free Campus, Rowland Hill Street, London NW3 2PF, United Kingdom; Tel.: +44 02074332853.
E-mail address: r.mookerjee@ucl.ac.uk (R.P. Mookerjee).
† These authors are joint first in their contribution
https://doi.org/10.1016/j.jhepr.2026.101922

autocrine sources of NE.[11] NE-activated macrophages result in downstream generation of pro-inflammatory cytokines, further amplifying an inflammatory cascade,[3] while also promoting and prolonging the activation of HSCs.[12] Studies have suggested that increased NE levels in sepsis modulate the responsiveness of macrophages to pro-inflammatory stimuli, through increased expression of the alpha-2a adrenergic receptor (ADRA2a), in turn perpetuating inflammation[13] and promoting hepatocellular dysfunction.[6,10]

ADRA2a is a member of the G-protein coupled receptor family and is abundant in many tissues, particularly within the reticuloendothelial system. On large arteries, ADRA2a maintains vessel tone, while in arterioles and venules, they paradoxically promote vasoconstriction.[14,15] It follows that selective antagonism of ADRA2A may increase systemic blood pressure while also enhancing blood flow through resistance vessels. Selective antagonism of ADRA2a in sepsis models has been shown to decrease liver injury and improve outcomes.[16]

Given that *in vitro* NE stimulates HSCs to proliferate and increase extracellular matrix production,[17,18] this raises the question of whether NE signals through ADRA2A expressed on HSCs and thereby influences fibrogenesis and portal hypertension. Therefore, this study aimed to examine the effects of ADRA2a on portal pressure in a rat model of severe portal hypertension; their role in promoting HSC activation; and finally, as to whether ADRA2a antagonism serves as a possible treatment for MASH in a diet-induced rat MASH model.

Materials and methods

ADRA2a antagonists

BRL44408 (Sigma-Aldrich, Dorset, UK), previously demonstrated to be a highly selectivity antagonist for the ADRA2a receptor sub-type,[19] was used for proof of concept in target studies for BDL portal hypertension pressure lowering, and for *in vitro* challenge of HSCs.

Yohimbine hydrochloride (YoHCl) (Sigma-Aldrich) also has good pharmacological selectivity for ADRA2a with no beta or alpha 1 receptor activity, and has been used clinically in therapy for male impotence and adjunctive diabetic therapy.[20] Given this, use of yohimbine repurposed for liver disease would be attractive if a beneficial effect was to be demonstrated.

Animals

Male Sprague Dawley rats (body weight 220-300 g; Charles River, UK) were housed in a temperature and light controlled (12 h light/dark cycle) facility at University College London as described in more detail in the supplementary methods.

BDL animal model: Rats underwent bile duct ligation (BDL) as previously described.[21] Sham-operated rats had a laparotomy without BDL. Four weeks after BDL or sham surgery, rats were allocated to one of four groups: (i) sham-operated (control) rats administered subcutaneous (sc) saline; (ii) BDL rats administered sc saline; (iii) BDL rats receiving sc BRL44408 (10 mg/kg, based on prior literature)[19,22] administered at 24 h and again 3 h prior to haemodynamic measurements; and (iv) sham-operated rats administered sc BRL44408 prior to haemodynamic measurements. Similarly, a cohort of BDL rats were also administered YoHCl [0.4 mg/kg based on prior literature[23] and our own dose ranging studies] for two doses, within 24 h of haemodynamic assessment as described in the supplemental methods.

MASH animal model: Twenty-four rats were fed normal chow (NC) (RM1; Special Diet Services, Witham, UK) or a high-fat, high-cholesterol (HFHC) diet (D09052204Y; Research Diets, USA) for 16 weeks as described previously.[24] For the final 8 weeks of the study, HFHC-fed rats were randomly allocated to receive YoHCl in their drinking water or control. Fluid was adjusted to achieve an average dose of 0.4 mg/kg/day. The animal groups were: (i) NC group: NC for 16 weeks (n = 9), (ii) HFHC group: HFHC diet for 16 weeks (n = 6) and (iii) HFHC+YoHCl group: HFHC diet and YoHCl for a final 8 weeks of study (n = 9).

Patients with MASLD

Fifteen patients with metabolic dysfunction-associated steatotic liver disease (MASLD) were recruited from the Virgen del Rocío University Hospital, Spain as described in the supplementary methods. Liver biopsies were obtained and mRNA expression of *ADRA2a* was evaluated using reverse-transcription quantitative PCR (RT-qPCR) as described in the supplementary methods.

HSCs

Cell isolation, culture, treatment, gel contraction and RT-qPCR

Primary human hepatic stellate cells (hHSCs) were isolated from wedge resection sections of human healthy liver tissue as previously described,[25] from patients undergoing surgery for primary liver cancer or metastasis from extrahepatic cancers at the Royal Free Hospital, London, UK after giving informed consent (REC reference[21]/WA/0388). Cells were isolated according to Mederacke *et al.*[26] with modifications for human liver[27] and cultured and treated with an ADRA2a agonist (guanfacine) or antagonist (BRL44408), as described in the supplementary methods. Gene expression of *ADRA2a* and *PDGFRβ* (platelet-derived growth factor receptor-β) in hHSCs was analysed by RT-qPCR, (described in supplemental methods).

Blood analyses

Plasma concentrations of alanine aminotransferase (ALT), aspartate aminotransferase (AST), albumin, bilirubin, and total cholesterol were measured using a Cobas Integra 400 automated analyser (Roche Diagnostics Ltd., Burgess Hill, UK).

Norepinephrine

Norepinephrine (NE) was extracted from plasma samples (EDTA), standards, and controls using a boronate affinity gel-coated plate. The extracted NE was acylated and enzymatically derivatised. The concentration of derivatised NE was measured using a competitive ELISA (LDN Labor Diagnostika Nord GmbH & Co. KG, Nordhorn, Germany) with a HRP-conjugated secondary antibody and TMB as substrate, and measured via a 96-well plate reader (BMG Labtech Ltd., UK).

FACS characterization of monocytes

The monocyte phagocytic-capacity was examined using FACS (described in the supplementary methods).

Liver tissue analyses

Liver histology

Formalin fixed liver tissue was processed and embedded in paraffin. 3-5 mm sections were de-waxed in xylene, stained with H&E or picrosirius red (PSR), and mounted in DPX. Slides were digitised using a NanoZoomer 2.0-HT digital slide scanner (Hamamatsu Photonics UK Ltd, Welwyn Garden City, UK). All histology specimens from MASH animals were reviewed by an experienced liver histopathologist (SHD) blinded to the clinical and laboratory data. NAFLD activity score was assessed using the Kleiner (NASH CRN) system.[28]

Collagen proportionate area measurement

Collagen proportionate area (CPA) was determined on NanoZoomer images of PSR-stained whole sections of liver as described in the supplementary methods.

Liver fat content quantification

Liver fat content was quantified using an adipogenesis detection assay kit (Abcam plc., UK) according to the manufacturer's instructions. Briefly, lipids were extracted from accurately weighed liver tissues, hydrolysed to free fatty acids and glycerol, and the released glycerol oxidised to generate colour from the probe, measured using a 96-well plate reader (BMG Labtech Ltd.).

Cyclic AMP analyses

Cyclic AMP in liver tissue was analysed using an ELISA technique. This was done by using 96-well plates pre-coated with mouse monoclonal anti-rabbit IgG, and blocked with a proprietary formulation of proteins (Cayman Chemical, Ann Arbor, MI, USA).

Western blot analysis

In BDL animals, hepatic protein expression of ADRA2a, caveolin-1, endothelial nitric oxide synthase (eNOS) and p-eNOS was measured using WB analysis as previously described[29] and in the supplementary methods.

RNA isolation and RT-qPCR

In MASH animals, mRNA expression of *ADRA2a* and *TIMP1* (TIMP metallopeptidase inhibitor 1) was determined using RT-qPCR (details in supplemental methods).

Hepatic MMP-2 protein expression

Protein expression of active matrix metalloproteinase-2 (MMP-2) in MASH animals was measured using gel zymography as described in the supplemental methods.

Hepatic protein expression of chemokines

In MASH animals, a rat cytokine and chemokine proteome profiler array (ARY008; Bio-Techne Ltd, Abingdon, UK) was used to screen 29 analytes, to determine the most relevant to be measured by ELISA. Hepatic protein expression of Ccl3 (macrophage inflammatory protein [MIP]-1α), Cx3cl1 (fractalkine) and Cxcl5 (LPS-induced CXC chemokine [LIX]) was then assessed by ELISA as described in the supplementary methods.

Hepatic CD68 protein expression

Liver sections (3–5 mm) from MASH animals were dewaxed in xylene and rehydrated through graded alcohols, and CD68 was detected by immunohistochemistry (IHC) as described in the supplementary methods.

Kupffer cell phagocytic function and ROS production

Isolation of liver non-parenchymal cells, the Kupffer cell phagocytic function and their reactive oxygen species (ROS) production were assessed as described in the supplementary methods.

Statistical analysis

Statistical analysis was performed using Prism 6 (GraphPad Software Inc., San Diego, CA, USA). The assumption of normality was checked using quantile-quantile plots (Q-Q plots) and histograms to ensure normally distributed variables. Differences between normally distributed variables were evaluated by ANOVA; when significant, *post hoc* tests were performed among groups by Student's *t* test. Some variables showed skewed distributions with variance heterogeneity and were analysed using Kruskal-Wallis rank test. When significant, *post hoc* tests were performed among groups by the Mann-Whitney test. Normally distributed data are presented as mean ± SD and non-parametric tested data are presented as median (IQR). p values <0.05 were considered statistically significant.

Results

ADRA2a plays a role in portal hypertension and hepatic inflammation

Study of a BDL animal model of significant portal hypertension

Body weight and liver biochemical parameters at termination are shown in Table 1. Following 24-hours treatment with the highly selective ADRA2a antagonist, BRL44408, there were no statistically significant changes in liver biochemical parameters. Evidence of chronic liver injury consistent with advanced fibrosis and nodule formation was demonstrated in the BDL rats compared with sham rats seen on H&E staining, as we have previously demonstrated[30] and no discernible histological changes in fibrosis were observed after BRL44408 treatment.

Hepatic expression of ADRA2a is increased in BDL cirrhosis

Expression of ADRA2A at both mRNA and protein levels was significantly increased in BDL rats compared with sham animals (p <0.01) (Fig. 1).

Haemodynamic derangement in BDL cirrhotic rats is reversed by ADRA2a antagonism

As previously demonstrated,[30] BDL rats demonstrated a marked increase in portal pressure compared to sham-operated rats (18 ± 4 *vs.* 8 ± 2 mmHg; p <0.0001), which was significantly reduced by BRL44408 (12 ± 3 mmHg; p

Table 1. Body weights and biochemical characteristics.

	Sham	BDL	BDL + BRL	*p* value, sham *vs.* BDL
Whole BW, end (g)	429 ± 62	391 ± 53	381 ± 48	0.11
ALT (U/L)	70 ± 9	100 ± 13	82 ± 6	<0.01
AST (U/L)	131 ± 17	445 ± 58	406 ± 65	<0.01
Albumin (g/L)	32.4 ± 0.8	24.4 ± 0.9	25.7 ± 1.0	<0.01
Bilirubin (µmol/L)	44 ± 15	132 ± 21	123 ± 12	<0.01

Whole BW and plasma concentrations of ALT, AST, albumin and bilirubin in sham animals, BDL animals and BDL animals treated with BRL44408 (BRL). Sham and BDL groups were compared using Student's *t*-test post-hoc after findig significant differences (p <0.05) comparing all three groups by one-way analysis of variance (ANOVA). Data are presented as mean ± SD. ALT, alanine aminotransferase; AST, aspartate aminotransferase; BDL, bile duct ligation; BW, body weight; BRL, BRL44408.

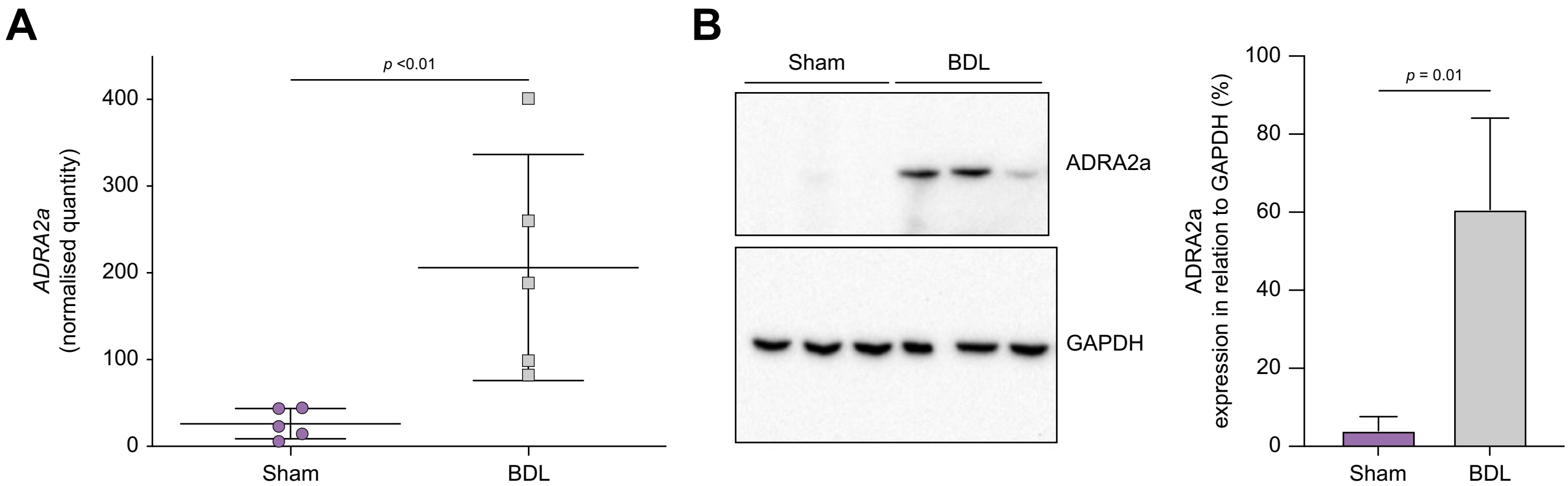

Fig. 1. Hepatic expression of ADRA2a. Sham rat livers have barely detectable expression of *ADRA2a* at mRNA (A) and protein levels (B). BDL surgery in rats after 4 weeks induces a marked increase in expression of ADRA2a. mRNA and protein expression of ADRA2a is normalized to GAPDH. Sham and BDL groups were compared using the Mann-Whitney test. BDL, bile duct ligation.

<0.0001) (Fig. 2A). Moreover, the BDL rats had lower mean arterial pressure (93 ± 13 *vs.* 110 ± 9 mmHg; p <0.01), which tended to increase in BDL rats post ADRA2a antagonism (102 ± 16 mmHg; p = 0.13) (Fig. 2B). The hepatic artery and portal venous blood flows were both reduced in the BDL rats when compared to sham animals (11 ± 6 *vs.* 24 ± 8 ml/min/kg BW; p <0.001 and 69 ± 19 *vs.* 179 ± 59 ml/min/kg BW; p <0.0001, respectively), and BRL44408 promoted a significant increase in both hepatic arterial blood flow (22 ± 8 ml/min/kg BW, p <0.01) and portal venous blood flow (93 ± 30 ml/min/kg BW; p = 0.05) (Fig. 2C,D).

We validated our findings of acute portal pressure reduction via ADRA2A antagonism using a second intervention, YoHCl, given its potential for direct clinical translation. BDL rats treated with YoHCl within 24 h also had a significant lowering of portal pressure compared to BDL rats injected with saline (10 ± 1 *vs.* 15 ± 2 mmHg, p <0.01).

Sympathetic nervous system activation in BDL rats

NE levels were markedly elevated in BDL rats compared to sham rats (194 ± 127 *vs.* 69 ± 29 pg/mL; p <0.01) (Fig. 3A). Furthermore, plasma NE was shown to correlate with increasing portal pressure (Pearson's r = 0.68; p <0.001) (Fig. 3B).

Changes in cyclic AMP levels in BDL rats reflecting NE signaling through ADRA2a

cAMP levels tended to decrease in BDL rats compared to sham rats (276.6 ± 16.5 *vs.* 318.7 ± 16.9 nmol/L; p = 0.10). However, after BRL44408, treatment of BDL rats, cAMP levels increased compared with BDL rats treated with saline (340.3 ± 15.8 *vs.* 276.6 ± 16.5 nmol/L; p = 0.01).

Impaired eNOS activity in cirrhosis is ameliorated by ADRA2a antagonism

eNOS activity was assessed by the p-eNOS/eNOS ratio. eNOS activity was decreased in BDL compared to sham rats, as demonstrated by a p-eNOS/eNOS ratio of 0.022 ± 0.007 *vs.* 0.710 ± 0.045. However, there was a restoration of eNOS phosphorylation towards sham levels in BRL44408-treated BDL rats, demonstrated by a p-eNOS/eNOS ratio of 0.373 ± 0.051 compared to untreated BDL rats. Furthermore, expression of caveolin-1, a known inhibitor of eNOS activity, was increased in BDL compared to sham rats, and was commensurately reduced after ADRA2a antagonism (Fig. 3C).

Markers of systemic and liver inflammation are decreased by ADRA2a antagonism

A detailed FACS characterization of monocytes revealed that the monocyte phagocytic-capacity was reduced in BDL rats compared with sham animals (31.3 ± 4.4 *vs.* 73.4 ± 4.8 mean fluorescence intensity [MFI]; p <0.001) and was restored towards normal levels in the BDL rats after treatment with BRL44408 (60.7 ± 6.3 MFI; p = 0.03). Moreover, in homogenized liver tissue, CD163/CD68 gating was used to define an activated Kupffer cell population, which was increased in BDL animals compared with sham animals (25.7 ± 1.5 *vs.* 15.5 ± 2.5 MFI; p = 0.01) (Fig. 3D). Following treatment with BRL44408,

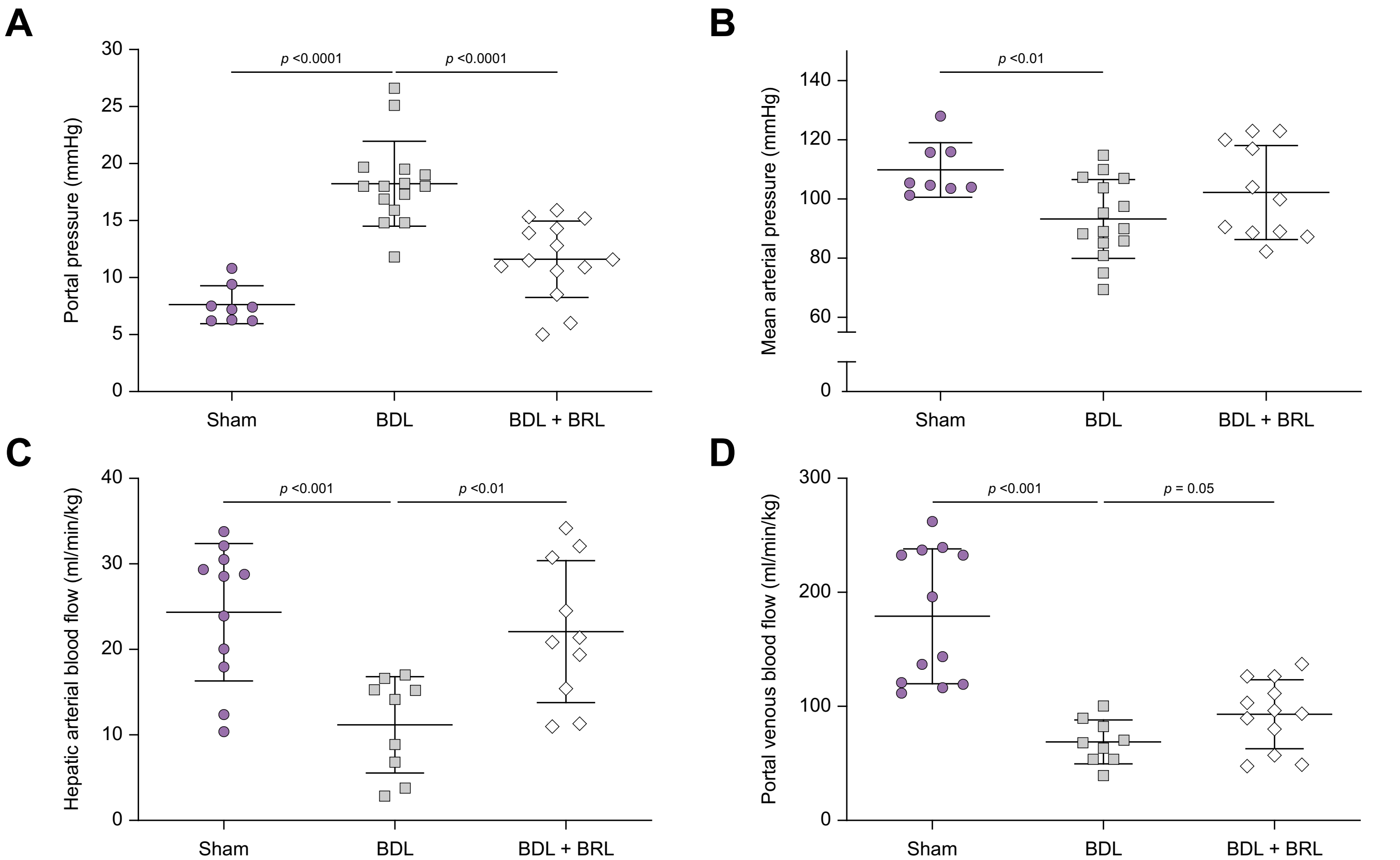

Fig. 2. Haemodynamics. Portal pressure is markedly increased in BDL rats compared to sham and ADRA2a antagonism with BRL44408 (BRL) significantly lowers portal pressure in BDL animals (A). Mean arterial blood pressure is significantly reduced in BDL rats and this is restored back towards sham levels with antagonism of ADRA2a (B). Hepatic arterial blood flow (C) and portal venous flow (D) is significantly reduced in BDL rats compared to sham and there is a significant increase in hepatic arterial flow and a modest increase in portal venous flow after ADRA2a antagonism. Differences between groups were analysed using Kruskal-Wallis rank test. When significant, *post hoc* tests were performed among groups by the Mann-Whitney test. BDL, bile duct ligation; BRL, BRL44408.

the number of active Kupffer cells in BDL rats was markedly reduced (16.7 ± 2.4 MFI; p <0.05). Aligned with the increase in activation status of Kupffer cells, generation of ROS by Kupffer cells also increased significantly in the BDL compared to sham rats (308.0 ± 17.3 *vs.* 105.8 ± 11.01 MFI; p <0.001) and was reduced in BDL rats following treatment with BRL44408 (79.6 ± 6.5 MFI; p <0.001) (Fig. 3E).

Liver tissue TNFα protein expression was markedly increased in BDL rats compared with sham rats (149.5 ± 73.7 *vs.* 55.3 ± 16.8 pg/ml; p <0.01); this was significantly decreased after BRL44408 therapy (93.1 ± 16.3 pg/ml; p = 0.03). Similarly, in portal venous blood, TNFα expression was increased in BDL rats (48.1 ± 4.1 *vs.* 20.1 ± 6.3 pg/ml; p <0.01) and reduced by BRL44408 treatment (31.1 ± 5.4 pg/ml; p = 0.03). Liver IL-6 expression was also markedly reduced after BRL44408 treatment compared with untreated BDL animals (14.4 ± 6.5 *vs.* 77.2 ± 14.8 pg/ml; p <0.01).

Isolated HSCs express ADRA2a and respond to ADRA2a agonism and antagonism

We determined whether hHSCs express ADRA2a by performing qRT-PCR on hHSCs obtained from 14 liver donors. We found detectable *ADRA2a* transcripts at varying levels in the different cell isolates. *PDGFRβ*, a transcript known to be expressed by hHSCs, was also confirmed to be expressed in all tested donors (Fig. 4A). To determine whether ADRA2a expression could lead to functional HSC activation as demonstrated by hHSC contractility, hHSCs were incubated in serum-free medium (SFM), SFM with an ADRA2a agonist (guanfacine), or SFM with guanfacine and the ADRA2a antagonist, BRL44408. Representative images from the cell collagen gel contraction assay and its quantification are shown in Fig 4B. Stimulation of the hHSCs with guanfacine was associated with a significant increase in gel contraction compared to SFM (47 ± 5 % *vs.* 71 ± 9; p <0.001), while the addition of the antagonist BRL44408 significantly attenuated guanfacine-induced HSC contraction (69 ± 3 %; p <0.001).

To determine whether these observations in hHSCs could also induce HSC contraction in rat liver, rat HSCs were incubated in SFM or guanfacine. Stimulation with guanfacine also led to an increase in the contraction of the 3D collagen gel compared to SFM, as was also seen with hHSCs (Fig. 4C).

ADRA2a expression is increased in patients with MASLD and evolving fibrosis

We investigated whether hepatic ADRA2a expression was increased in patients with MASLD fibrosis. In patients with MASLD and established F1-3 fibrosis, the hepatic mRNA expression of *ADRA2a* was increased compared to that in patients with F0 fibrosis (90.9 ± 81.6 *vs.* 19.4 ± 16.4). The clinical, biochemical, and histological characteristics of the two groups are shown in Table S1.

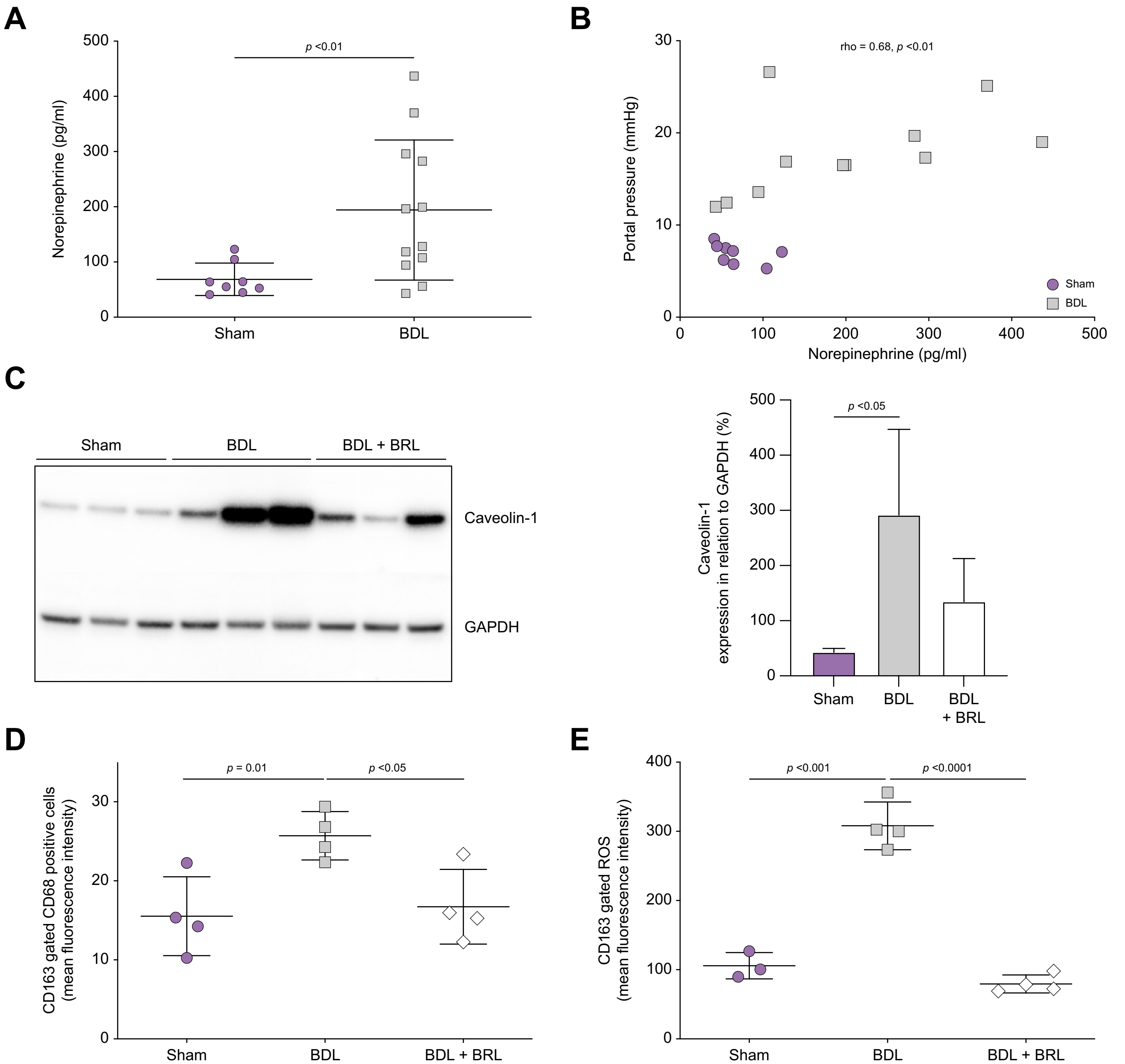

Fig. 3. Sympathetic nervous system, caveolin-1 and markers of inflammation. Norepinephrine levels are markedly elevated in BDL rats compared to sham (A) (the Mann-Whitney test) and correlate with increasing portal pressure (B) (Pearson correlation test). Hepatic expression of caveolin-1, a known inhibitor of eNOS activity is increased in BDL rats compared to sham and reduced after BRL treatment (C) (the Mann-Whitney test). In liver tissue, numbers of activated Kupffer cells were increased in BDL animals compared to sham animals and markedly reduced following BRL treatment (D) (one-way ANOVA and Student's *t* test). The generation of ROS by Kupffer cells also increased significantly in the BDL rats compared to sham and was reduced following BRL44408 treatment (E) (one-way ANOVA and Student's *t* test). BDL, bile duct ligation; BRL, BRL44408; ROS, reactive oxygen species.

Antagonism of ADRA2a decreases fibrosis and inflammation in MASH

Study of a MASH animal model of fibrosis

Table 2 describes the clinical, biochemical and histological differences between HFHC-fed animals and those on NC, as well as the impact of ADRA2a antagonism. No differences in body weight increments were observed between the groups after 16 weeks. However, over the final 8 weeks of intervention, HFHC-fed rats gained less weight than NC-fed animals. By contrast, YoHCl-treated animals on a HFHC diet had growth not dissimilar to NC. Of note, there were no differences in the kilo calories consumed between the three groups.

HFHC-fed rats had increased liver weight and a significantly higher liver-to-body weight ratio than NC-fed rats; both measures were significantly reduced by YoHCl treatment compared with untreated HFHC-fed rats. Plasma cholesterol, ALT and AST levels were increased in HFHC- compared to NC-fed rats, and YoHCl treatment significantly reduced AST levels but had less impact on ALT.

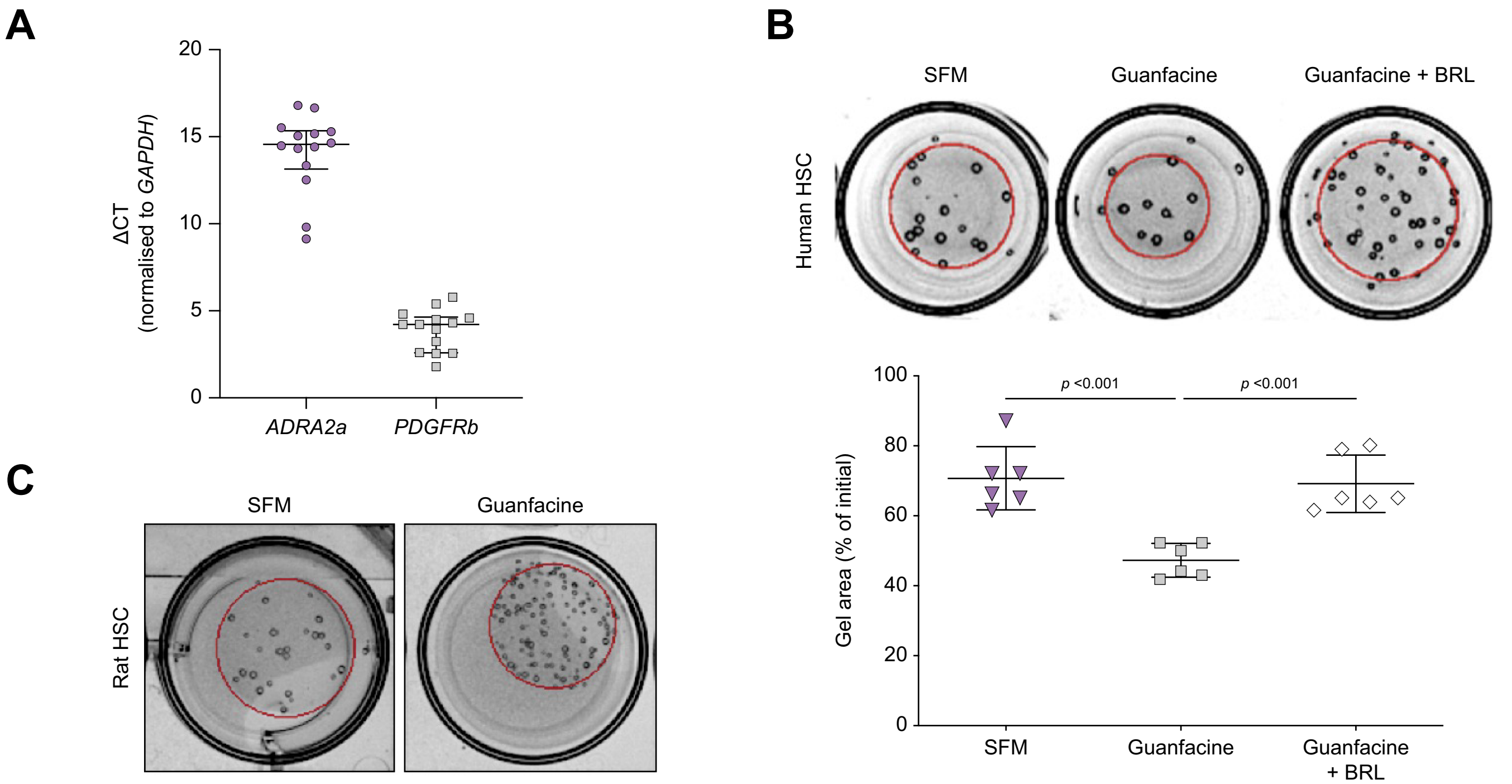

Fig. 4. Expression of ADRA2a and contractility of HSCs. Human HSCs express both ADRA2a and PDGFRβ to varying levels amongst the 14 donor livers tested (A). Human HSCs grown in the presence of guanfacine show significantly greater contraction in a 3D collagen gel than cells grown in SFM in the absence of guanfacine. Cells grown in the presence of both guanfacine and BRL44408 show less contraction in a 3D collagen gel than cells grown in the presence of guanfacine alone (B) (one-way ANOVA and Student's *t* test). Rat HSCs grown in the presence of guanfacine show greater contraction in a 3D collagen gel than cells grown in the absence of guanfacine (C). Of note, the black dots in the gel contraction areas represent artefacts (air bubbles). BRL, BRL44408; HSCs, hepatic stellate cells; SFM, serum-free medium.

Table 2. Weights, biochemical characteristics and histological scores of liver tissue.

	Normal chow	HFHC diet	HFHC diet + YoHCl	*p* value	*p* value	*p* value
	n = 9	n = 6	n = 9	All 3 groups	NC *vs.* HFHC	HFHC *vs.* HFHC+Yo
Whole BW, start (g)	305 ± 26	309 ± 14	305 ± 16	0.91		
Whole BW, end (g)	591 ± 31	559 ± 41	581 ± 49	0.33		
Weight gain (g)	286 ± 31	250 ± 39	276 ± 38	0.16		
Weight gain (g) final 8 weeks	89 ± 17	53 ± 24	79 ± 20	0.01	<0.01	0.03
Liver weight (g)	16.4 ± 2.0	36.3 ± 5.2	33.5 ± 3.3	<0.0001	<0.0001	0.22
Liver/body weight (%)	2.8 ± 0.3	6.5 ± 0.9	5.8 ± 0.4	<0.0001	<0.0001	<0.05
Liver fat (µmol/mg liver)	17 (12-22)	41 (26-90)	29 (23-40)	<0.01	<0.01	0.39
Total cholesterol (mmol/L)	1.2 (1.1-1.5)	3.7 (2.7-4.5)	2.8 (2.4-3.5)	<0.001	<0.001	0.24
ALT (U/L)	40 (37-50)	142 (96-190)	113 (87-162)	<0.001	<0.001	0.49
AST (U/L)	100 (92-129)	329 (312-356)	260 (230-292)	0.0001	<0.001	0.02
Steatosis	0 ± 0	3 ± 3	3 ± 3	<0.0001	<0.0001	1.0
Inflammation	0.1 ± 0.3	1.0 ± 0.0	0.8 ± 0.8	0.01	0.001	0.53
Ballooning	0 ± 0	1.8 ± 0.4	0.6 ± 0.5	<0.0001	0.001	<0.01
NAFLD activity score	0.1 ± 0.3	5.8 ± 0.4	4.3 ± 1.1	<0.0001	0.001	<0.01
Fibrosis	0 ± 0	2.6 ± 1.1	1.1 ± 0.9	<0.0001	<0.0001	0.02

Whole BW, weight gain, and liver weight, liver fat concentration and plasma concentrations of total cholesterol, ALT, and AST and histological scores of liver tissue (steatosis, inflammation, ballooning, NAFLD activity score and fibrosis) in control animals (n = 9), HFHC-fed animals (n = 6) and HFHC-fed animals treated with YoHCl for the final 8 weeks of the 16-week study (n = 9). Normally distributed variables were analysed using one-way ANOVA and data showing skewed distributions and variance heterogeneity with Kruskal-Wallis rank test. When significant, *post hoc* tests were performed among groups by Student's *t* test and the Mann-Whitney test, respectively. Data tested using parametric tests are presented as mean ± SD. Data tested using non-parametric tests are presented as median (IQR). ALT, alanine aminotransferase; AST, aspartate aminotransferase; BDL, bile duct ligation; BW, body weight; HFHC, high-fat high-cholesterol; YoHCl, yohimbine hydrochloride.

Microscopically, HFHC rat livers showed severe steatosis, with varying degrees of hepatocyte ballooning, inflammation and fibrosis. Overall, the HFHC-fed animals showed histological evidence of MASH; HFHC livers showed areas of perisinusoidal, periportal and bridging fibrosis. YoHCl treatment decreased the NAFLD activity score and degree of ballooning, in addition to reducing histological features of fibrosis compared to non-treated HFHC-fed rats (1.1 ± 0.9 *vs.* 2.6 ± 1.1; $p = 0.02$). As expected, livers from NC-fed animals had normal histology.

Liver fat content was quantified by measuring the mono-, di-, and triglycerides extracted from liver tissue. Liver fat content was significantly higher in HFHC-fed rats than NC-fed rats, and did not change significantly following YoHCl therapy (Table 2).

ADRA2a expression is increased in MASH rats

ADRA2a was significantly overexpressed in the livers of HFHC-compared to NC-fed animals (90.9 ± 33.2 *vs.* 36.6 ± 25.1; p = 0.001), and this expression was not changed by YoHCl treatment (73.3 ± 23.3; p = 0.23).

ADRA2a antagonism reduces progression of hepatic fibrosis in MASH rats

To quantify degree of fibrosis, CPA was measured by digital image analysis of PSR staining, as shown in Fig. 5A. CPA increased in HFHC-fed compared to NC-fed animals (15.0 (13.0-19.5) *vs.* 1.4 (0.6-1.5), p <0.01) and YoHCl treatment significantly reduced this (7.6 (4.1-9.2); p <0.01) (Fig. 5B).

The gene expression of TIMP1, the main regulator of extracellular matrix composition, was increased in HFHC-compared to NC-fed animals and significantly reduced by YoHCl (8.4 (6.1-9.2) *vs.* 13.5 (9.2-25.8), p = 0.03) (Fig. 5C). However, as fibrogenesis involves rearrangement as well as synthesis of collagen fibres, we also assessed MMP activity, as demonstrated by active MMP-2. Protein expression of active MMP-2 increased significantly in HFHC- compared to NC-fed animals (16.6 (10.5-25.3) *vs.* 3.8 (2.9-7.6); p = 0.04) and was reduced by YoHCl (5.6 (4.8-9.8), p = 0.05) (Fig. 5D), supporting the reduction in fibrosis seen on CPA analysis.

ADRA2a antagonism reduces hepatic inflammation in MASH rats

To determine the most relevant cytokines and/or chemokines to investigate in HFHC rat liver, a pilot study using a proteome profiler array was performed (data not shown). This identified three cytokines and chemokines that were selected for further study: Ccl3 (MIP-1α), Cx3cl1 (fractalkine) and Cxcl5 (LIX). Protein expression of Ccl3 was increased in HFHC- compared to NC-fed animals (1,425 ± 278 *vs.* 365 ± 192; p <0.0001) and reduced by YoHCl (711 ± 196; p <0.0001). Similarly, protein expression of Cx3cl1 was increased in HFHC- compared to NC-fed animals (1256 ± 225 *vs.* 809 ± 114; p <0.0001) and reduced by YoHCl (955 ± 112; p <0.001). Finally, Cxcl5 protein expression was also increased in HFHC- compared to NC-fed animals (158 ± 35 *vs.* 131 ± 11); p = 0.01) and reduced by YoHCl (135 ± 11; p = 0.03) (Fig. 6A).

Moreover, in liver tissue, CD68 staining demonstrated increased numbers of resident macrophages and infiltrating monocytes/macrophages in HFHC- compared to NC-fed animals (3.92 ± 1.02 *vs.* 0.70 ± 0.40, p <0.0001). Treatment with YoHCl resulted in decreased numbers of CD68-positive cells

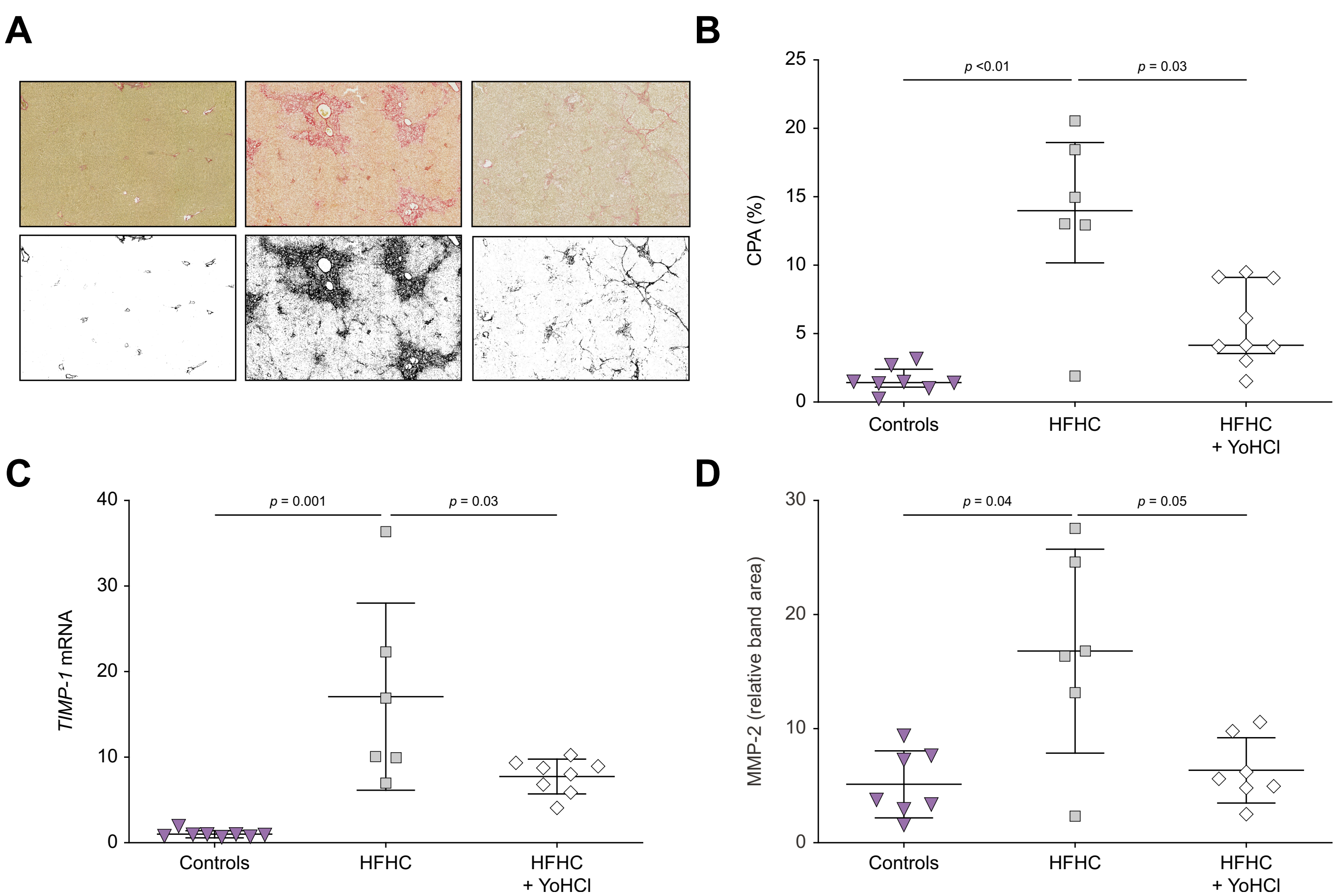

Fig. 5. Hepatic fibrosis in MASH rats. CPA is increased in HFHC-induced MASH animals compared to controls and significantly reduced by YoHCl treatment, as shown by representative picrosirius red staining and CPA quantification of the liver (A, B). Hepatic gene expression *TIMP1* (C) and protein expression of active MMP-2 (D) are increased in HFHC-fed animals and significantly reduced by YoHCl. Differences between groups were analysed using Kruskal-Wallis rank test. When significant, *post hoc* tests were performed among groups by the Mann-Whitney test. CPA, collagen proportionate area; HFHC, high-fat high-cholesterol; MASH, metabolic dysfunction-associated steatohepatitis; YoHCl, yohimbine hydrochloride.

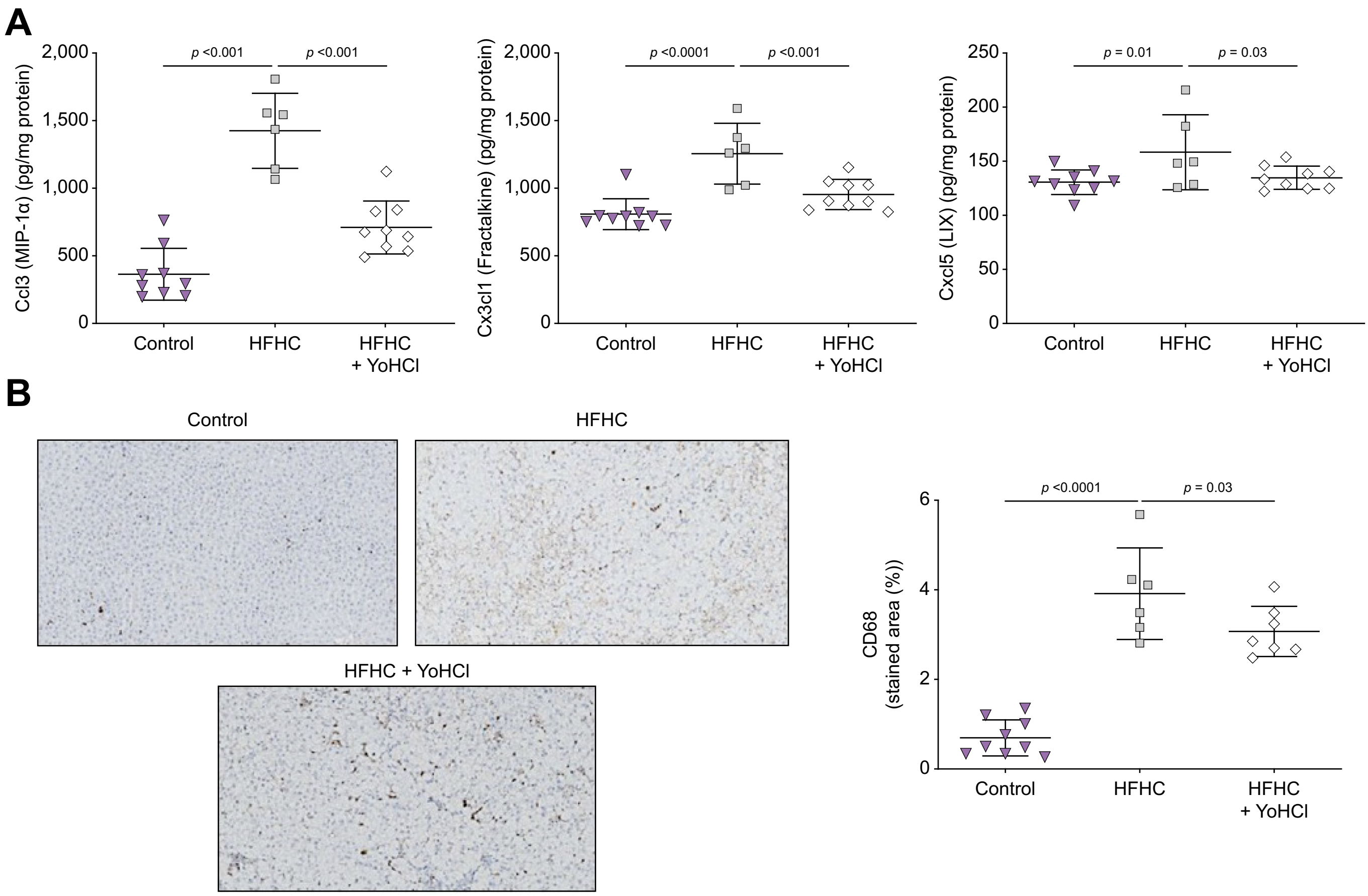

Fig. 6. Hepatic inflammation in MASH rats. The increased hepatic protein expression of Ccl3 (macrophage inflammatory protein [MIP]-1α), Cx3cl1 (fractalkine) and Cxcl5 (LPS-induced CXC chemokine [LIX]) in HFHC-induced MASH rats are reduced by YoHCl (A). Likewise, YoHCl reduces the increased number of CD68-positive cells in the liver tissue. A representative CD68 immunohistochemical stain of liver tissue is shown (B). Differences between groups were analysed using Kruskal-Wallis rank test. When significant, *post hoc* tests were performed among groups by the Mann-Whitney test. HFHC, high-fat high-cholesterol; MASH, metabolic dysfunction-associated steatohepatitis; YoHCl, yohimbine hydrochloride.

(3.07 ± 0.56, p = 0.03) (Fig. 6B), supporting the reduction in hepatic inflammation.

Discussion

In this study, we show that severity of portal hypertension in BDL cirrhotic rats correlates with NE concentrations and an increased expression of ADRA2a, whilst treatment with two different ADRA2a antagonists, namely BRL44408 and YoHCl, significantly lowers portal pressure. In addition, we show increased liver blood flow and preserved mean arterial pressure, suggesting a likely impact on intrahepatic resistance and an improvement in overall systemic haemodynamics with ADRA2a antagonism. This contrasts with current clinical treatment with beta-blockade which often impacts on the systemic circulation, lowering mean arterial pressure and liver blood flow, which may aggravate cirrhosis decompensation.[31]

Sympathetic overactivation has been associated with the progression of cirrhosis to decompensation,[32] while plasma NE levels have been reported to correlate with the severity of portal hypertension, systemic inflammation, and clinical outcomes in cirrhosis.[8,9,33] Increased sympathetic tone (NE), along with reduced vasodilatory factors, drives an increase in intrahepatic resistance in cirrhosis, compounded further by inflammation as cirrhosis progresses.[1,34] At a mechanistic level, activation and contraction of HSCs, often in the context of hepatic inflammation, in conjunction with reduced liver generation of endothelial NO, are key elements behind an increase in hepatic vascular resistance.[35–37]

Given this, we assessed the impact on hepatic NO bioavailability and demonstrated that ADRA2a antagonism helps restore eNOS activity in BDL cirrhosis rats and reduces caveolin-1 expression (an inhibitor of eNOS). In addition, we show that inhibiting ADRA2a lowers systemic and hepatic inflammation, with reduced ROS generation and activation of Kupffer cells. This may, in part, explain the rapid reduction in portal pressure within 24 h post both BRL44408 and YoHCl treatment of BDL rats, as noted with other interventions that reduce eNOS inhibition and lower hepatic inflammation.[38,39]

To date, treatment of portal hypertension focusses largely on NSBBs to induce portal pressure reduction in about 40-50% of cases, largely by reducing portal inflow. However, NSBBs are associated with reduction in cardiac output from action on cardiac beta-1 receptors and reduced organ perfusion (including hepatic), which is most marked as cirrhosis progresses. More recently, agents such as carvedilol, also combining alpha 1-adrenergic receptor antagonism with beta-blockade, enhance portal pressure lowering but again

negatively impact systemic haemodynamics. ADRA2a subtype receptors are reported to have distinct effects: their central activation decreases systemic blood pressure, in part by promoting NO release,[14] whereas in small resistance vessels they induce vasoconstriction.[15] This provided the rationale for antagonizing ADRA2a in the context of portal hypertension: to increase systemic blood pressure and improve blood flow through resistance vessels. These effects were indeed observed in our BDL cirrhosis model, where both hepatic arterial and portal venous components of liver blood flow improved following treatment with the highly selective ADRA2a antagonist BRL44408, which also reduced portal pressure while maintaining mean arterial pressure.

HSCs become activated during liver injury, such as in MASH and alcohol-related steatohepatitis, and undergo myofibroblastic transformation, whereby contractile elements modulate the tonicity of neighboring liver resistance vasculature.[35] Inflammatory signals from activated innate immune cells, in addition to hepatocyte factors generated during liver injury, promote HSC activation.[40,41] Moreover, activated HSCs in turn produce pro-fibrogenic factors and inflammatory cytokines, further adding to liver injury progression.[42] In this study, we demonstrate increased activation of HSCs in patients and in rat primary HSCs upon stimulation with a selective ADRA2a agonist, guanfacine, which can be reversed by the addition of the highly selective ADRA2a antagonist BRL44408, with a functional reduction in contractility. This may provide a further mechanistic explanation for the reduction in portal pressure in BDL rats after ADRA2a antagonism.

In addition to its contribution to portal hypertension, HSC activation plays a key role in fibrogenesis. *In vitro*, adrenergic receptor antagonism reduces extracellular matrix production in a mouse HSC cell line[43] and inhibits proliferation in mouse and rat primary HSCs.[17] Moreover, chemical sympathectomy in a carbon tetrachloride rat model, ameliorates liver fibrogenesis, highlighting the importance of noradrenergic signaling in the development of fibrosis.[44] In this study, reducing HSC contractility with ADRA2a antagonism is associated with improved cAMP generation, reflecting downstream signaling through the G-protein αi-subunit of ADRA2a.[45] How NE acts on HSCs and contributes to liver disease progression has not been extensively explored to date; however, our data suggest a potential mechanism involving ADRA2a, as evidenced by *in vitro* effects on HSC activation, where selective ADRA2a antagonism decreased fibrogenic markers (TIMP-1 and MMP2 activity) and reduced CPA in our MASH model after 8 weeks of YoHCl treatment. This requires further elaboration in other fibrosis models.

ADRA2a is expressed in high concentrations in the mesenteric vascular bed and on immune cells, particularly in the reticulo-endothelial system. Our data suggest that hepatic ADRA2a expression is upregulated in the rodent models of liver disease we studied and aligns with literature in sepsis models, where antagonism of ADRA2a deactivates inflammatory pathways in Kupffer cells.[22] Moreover, our data supports the contention that in the setting of high NE levels in liver disease, increased ADRA2a expression on Kupffer cells[10,13] allows for an autocrine pro-inflammatory increase in their activation.[6] HSCs are then activated either indirectly through inflammatory cascades, or directly via ADRA2a upregulation on HSCs. This might be expected to further aggravate intrahepatic resistance, while also promoting fibrosis progression. An important point to note, previously described in the literature as a consequence of ADRA2a antagonism, is a slight increase in plasma NE levels.[46] This has been attributed to a block on central pre-synaptic feedback inhibition of NE release. In spite of this, we show there is reduced inflammation and improved haemodynamics, suggesting potential positive effects downstream of ADRA2a signalling.

Inflammation is a major driver of fibrosis progression in liver disease and subsequent cirrhosis decompensation and poor outcome,[47,48] while elevated NE signalling has also been implicated in organ injury in inflammatory models.[16] ADRA2a antagonism reduces inflammation and cytokine production and improves survival when administered after the onset of sepsis in such models.[13] Thus, the beneficial effects of ADRA2a antagonism in liver disease are likely pleiotropic, involving both direct effects on HSCs and Kupffer cell activation, as well as indirect effects through modulation of the inflammatory hepatic milieu, as demonstrated by reduced CD68+ infiltrating cells and decreased Ccl3 and Cxcl5 chemokines in the livers of MASH animals treated with YoHCl, alongside reduced fibrogenesis. This could not only impact on fibrosis progression but clearly also on mechanisms that perpetuate portal hypertension, including inhibition of eNOS. Whether this impacts wider organ dysfunction associated with cirrhosis decompensation remains to be elucidated.

While we have focused on the role of ADRA2a on HSCs and others have described the role of ADRA2a on Kupffer cells, it is possible that ADRA2a also impacts other liver cell types. Whilst this remains a subject for future investigation, we believe it is unlikely to impact upon our understanding of the relative translational contribution of ADRA2a to fibrogenesis or portal hypertension, as investigated in this study.

A further limitation was the practicality of BRL44408 as a tool to address long-term, highly selective antagonism of ADRA2a in our MASH model, given its prohibitive costs and limited availability. However, YoHCl has a relative high selectivity for ADRA2a (with no activity on alpha-1 and beta receptors) and importantly, not only recapitulates the significant acute portal pressure reduction in BDL rats seen with BRL44408 but also reduces inflammation and fibrosis after 8 weeks treatment in our MASH model. Given YoHCl has a good safety profile and is used for other non-hepatological indications in humans, we considered this a good agent to evaluate, given opportunities for future translational studies in patients with liver disease.

Only one model was used for the portal hypertension element of this study. The BDL rat enables evaluation of a sustained inflammatory response with advancing portal hypertension, until extrahepatic organ injury ensues. This emulates the hyperdynamic circulation in patients with advanced cirrhosis, during which the autonomic system undergoes further derangement.[49] The HFHC MASH model was chosen as the most relevant for human translation, showing fibrosis and hepatic inflammation without the introduction of a toxic cofounder, *e.g.* thioacetamide or carbon tetrachloride.[50] The impact of such toxins on metabolic pathways, and specifically mitochondrial function, would make subsequent evaluation of potential mechanisms translatable to humans difficult. By contrast, the innate inflammatory response observed in both BDL and MASH models – likely driven in part by ADRA2a

signaling and contributing to inflammatory injury as previously described[6] – supports both their translational relevance and our choice of models.

We also acknowledge the limitation of no direct comparison between selective ADRA2a antagonism in the BDL model of portal hypertension and agents used in standard clinical care such as propranolol and carvedilol. In keeping with the principles of Replacement, Reduction and Refinement in animal research, our ethics committee advised against this. Whilst propranolol and carvedilol both lower portal pressure, both agents are well known to induce systemic hypotension and reduce liver blood flow, which we show is not the case for selective ADRA2a antagonism. Clearly, a future study of ADRA2a antagonism in patients with cirrhosis would be important to further elaborate on the results of this study.

In conclusion, our study shows that ADRA2a expression is increased in two different models of liver disease and in patients with MASH fibrosis. We demonstrate a role for ADRA2a in the pathogenesis of experimental portal hypertension and fibrogenesis and suggest this occurs through modulation of liver inflammation and HSC activation, as demonstrated by the effects of two different selective ADRA2a antagonists. These data suggest ADRA2a antagonism should be explored further as a potential target for therapeutic intervention in the management of portal hypertension and fibrosis progression.

Affiliations

[1]UCL Institute of Liver and Digestive Health, University College London, United Kingdom; [2]Department of Hepatology & Gastroenterology, Aarhus University Hospital, Denmark; [3]SeLiver Group, Instituto de Biomedicina de Sevilla (HUVR/CSIC/US), Hepatic and Digestive Diseases Networking Biomedical Research Centre (CIBERehd), Digestive Diseases Unit, Hospital Universitario Virgen del Rocío, Department of Medicine, University of Seville, Sevilla, Spain; [4]Institute of Pathology, Aarhus University Hospital, Denmark

Abbreviations

ADRA2A, alpha 2A adrenergic receptor; ALT, alanine aminotransferase; AST, aspartate aminotransferase; BDL, bile duct ligation; CPA, collagen proportionate area; eNOS, endothelial nitric oxide synthase; HFHC, high-fat, high-cholesterol; HSCs, hepatic stellate cells; IHC, immunohistochemistry; MASLD, metabolic dysfunction-associated steatotic liver disease; MASH, metabolic dysfunction-associated steatohepatitis; MMP-2, matrix metalloproteinase-2; NE, norepinephrine; NC, normal chow; NSBB, non-selective beta-blockade; PDGFRβ, platelet-derived growth factor receptor-β; PSR, picrosirius red; ROS, reactive oxygen species; RT-qPCR, reverse transcription quantitative real-time polymerase chain reaction; SFM, serum-free medium; TIMP1, TIMP metallopeptidase inhibitor 1; YoHCl, yohimbine hydrochloride.

Financial support

RPM received grants from Higher Education Funding UK (HEIF) and also early proof of concept funding from UCL Business (POC-12-044). KLT was supported by grants from the Danish Council for Independent Research (DFF-6110-00536) and Novo Nordic Foundation (NFF19OC0055039).

Conflicts of interest

Manuel Romero-Gómez provided services of consulting for Abbvie, Alphasigma, Advanz, Apollo, Astra-Zeneca, Bausch Health, BMS, Boehringer-Ingelheim, Exo-Biologics, Gilead, Ipsen, MSD, Novo-Nordisk, Pfizer, Prosciento, Resolution Therapeutics, Roche, Rubió, Sagimet, Siemens, UCB pharma, and got research Grants from Gilead, Intercept, Siemens, Theratechnologies, Novo-Nordisk, Echosens. Krista Rombouts ownes shares or received stock options and receives consultancies in Engitix Therapeutics Ltd. Rajiv Jalan is founder of Yaqrit Limited, a spin out company from University College London. He also is co-founder of Hepyx Limited and Cyberliver Limited. Rajeshwar Prosad Mookerjee is a co-founder of Yaqrit Limited, and also has scientific collabaorations with Cyberliver Limited and Hepyx Limited. He is co-inventor of patents filed by UCL Business around ADRA2a, now licensed to Hepyx Limited. All other authors have no conflicts to declare.

Please refer to the accompanying ICMJE disclosure forms for further details.

Authors' contributions

RPM conceived and designed the study, and sought funding for the project. VS, HJ, AH, MCM and ND carried out the experiments. VS, HJ, ACM, and SEA analysed the samples. RGD and MRG contributed cDNA for analysis. KLT, VS, HJ, SHD and RPM analysed and interpreted the data. KLT, VS, HJ, ACM and RPM drafted the manuscript. KLT, RJ, KR and RPM critically revised the manuscript for important intellectual content.

Supplementary data

Supplementary data to this article can be found online at https://doi.org/10.1016/j.jhepr.2026.101922.

References

[1] Iwakiri Y, Shah V, Rockey DC. Vascular pathobiology in chronic liver disease and cirrhosis - current status and future directions. J Hepatol 2014;61(4):912–924.

[2] Reiberger T, Ulbrich G, Ferlitsch A, et al. Carvedilol for primary prophylaxis of variceal bleeding in cirrhotic patients with haemodynamic non-response to propranolol. Gut 2013;62(11):1634–1641.

[3] Francque S, Verrijken A, Mertens I, et al. Noncirrhotic human nonalcoholic fatty liver disease induces portal hypertension in relation to the histological degree of steatosis. Eur J Gastroenterol Hepatol 2010;22(12):1449–1457.

[4] Ekstedt M, Hagstrom H, Nasr P, et al. Fibrosis stage is the strongest predictor for disease-specific mortality in NAFLD after up to 33 years of follow-up. Hepatology 2015;61(5):1547–1554.

[5] Gracia-Sancho J, Marrone G, Fernandez-Iglesias A. Hepatic microcirculation and mechanisms of portal hypertension. Nat Rev Gastroenterol Hepatol 2019;16(4):221–234.

[6] Flierl MA, Rittirsch D, Nadeau BA, et al. Upregulation of phagocyte-derived catecholamines augments the acute inflammatory response. PloS one 2009;4(2):e4414.

[7] Stadlbauer V, Wright GA, Banaji M, et al. Relationship between activation of the sympathetic nervous system and renal blood flow autoregulation in cirrhosis. Gastroenterology 2008;134(1):111–119.

[8] Mehta G, Mookerjee RP, Sharma V, et al. Systemic inflammation is associated with increased intrahepatic resistance and mortality in alcohol-related acute-on-chronic liver failure. Liver Int 2015;35(3):724–734.

[9] Weiss E, de la Pena-Ramirez C, Aguilar F, et al. Sympathetic nervous activation, mitochondrial dysfunction and outcome in acutely decompensated cirrhosis: the metabolomic prognostic models (CLIF-C MET). Gut 2023;72(8):1581–1591.

[10] Zhou M, Yang S, Koo DJ, et al. The role of Kupffer cell alpha(2)-adrenoceptors in norepinephrine-induced TNF-alpha production. Biochim Biophys Acta 2001;1537(1):49–57.

[11] Yang S, Koo DJ, Zhou M, et al. Gut-derived norepinephrine plays a critical role in producing hepatocellular dysfunction during early sepsis. Am J Physiol Gastrointest Liver Physiol 2000;279(6):G1274–G1281.

[12] Pradere JP, Kluwe J, De Minicis S, et al. Hepatic macrophages but not dendritic cells contribute to liver fibrosis by promoting the survival of activated hepatic stellate cells in mice. Hepatology 2013;58(4):1461–1473.

[13] Miksa M, Das P, Zhou M, et al. Pivotal role of the alpha(2A)-adrenoceptor in producing inflammation and organ injury in a rat model of sepsis. PLoS ONE 2009;4(5):e5504.

[14] Shafaroudi MM, McBride M, Deighan C, et al. Two "knockout" mouse models demonstrate that aortic vasodilatation is mediated via alpha2a-adrenoceptors located on the endothelium. J Pharmacol Exp Ther 2005;314(2):804–810.

[15] Duka I, Gavras I, Johns C, et al. Role of the postsynaptic alpha(2)-adrenergic receptor subtypes in catecholamine-induced vasoconstriction. Gen Pharmacol 2000;34(2):101–106.

[16] Yang S, Zhou M, Chaudry IH, et al. Norepinephrine-induced hepatocellular dysfunction in early sepsis is mediated by activation of alpha2-adrenoceptors. Am J Physiol Gastrointest Liver Physiol 2001;281(4):G1014–G1021.
[17] Oben JA, Roskams T, Yang S, et al. Hepatic fibrogenesis requires sympathetic neurotransmitters. Gut 2004;53(3):438–445.
[18] Tang WC, Chang YW, Che M, et al. Thioacetamide-induced norepinephrine production by hepatocytes is associated with hepatic stellate cell activation and liver fibrosis. Curr Mol Pharmacol 2022;15(2):454–461.
[19] Dwyer JM, Platt BJ, Rizzo SJ, et al. Preclinical characterization of BRL 44408: antidepressant- and analgesic-like activity through selective alpha2A-adrenoceptor antagonism. Int J Neuropsychopharmacol 2010;13(9):1193–1205.
[20] Tang Y, Axelsson AS, Spegel P, et al. Genotype-based treatment of type 2 diabetes with an alpha2A-adrenergic receptor antagonist. Sci translational Med 2014;6(257). 257ra139.
[21] Poo JL, Estanes A, Pedraza-Chaverri J, et al. [Chronology of portal hypertension, decreased sodium excretion, and activation of the renin-angiotensin system in experimental biliary cirrhosis]. Rev Invest Clin 1997;49(1):15–23.
[22] Zhang F, Wu R, Qiang X, et al. Antagonism of alpha2A-adrenoceptor: a novel approach to inhibit inflammatory responses in sepsis. J Mol Med (Berlin, Germany) 2010;88(3):289–296.
[23] Wang Y, Yu X, Wang F, et al. Yohimbine promotes cardiac NE release and prevents LPS-induced cardiac dysfunction via blockade of presynaptic alpha2A-adrenergic receptor. PLoS ONE 2013;8(5):e63622.
[24] Thomsen KL, Gronbaek H, Glavind E, et al. Experimental nonalcoholic steatohepatitis compromises ureagenesis, an essential hepatic metabolic function. Am J Physiol Gastrointest Liver Physiol 2014;307(3):G295–G301.
[25] Caon E, Martins M, Hodgetts H, et al. Exploring the impact of the PNPLA3 I148M variant on primary human hepatic stellate cells using 3D extracellular matrix models. J Hepatol 2024;80(6):941–956.
[26] Mederacke I, Dapito DH, Affo S, et al. High-yield and high-purity isolation of hepatic stellate cells from normal and fibrotic mouse livers. Nat Protoc 2015;10(2):305–315.
[27] Rombouts K, Carloni V. Determination and characterization of tetraspanin-associated phosphoinositide-4 kinases in primary and neoplastic liver cells. Methods Mol Biol 2016;1376:203–212.
[28] Kleiner DE, Brunt EM, Van Natta M, et al. Design and validation of a histological scoring system for nonalcoholic fatty liver disease. Hepatology 2005;41(6):1313–1321.
[29] Jalan R, De Chiara F, Balasubramaniyan V, et al. Ammonia produces pathological changes in human hepatic stellate cells and is a target for therapy of portal hypertension. J Hepatol 2016;64(4):823–833.
[30] Balasubramanian V, Mehta G, Jones H, et al. Post-transcriptional regulation of hepatic DDAH1 with TNF blockade leads to improved eNOS function and reduced portal pressure in cirrhotic rats. Scientific Rep 2017;7(1):17900.
[31] Serste T, Francoz C, Durand F, et al. Beta-blockers cause paracentesis-induced circulatory dysfunction in patients with cirrhosis and refractory ascites: a cross-over study. J Hepatol 2011;55(4):794–799.
[32] Henriksen JH, Ring-Larsen H, Kanstrup IL, et al. Splanchnic and renal elimination and release of catecholamines in cirrhosis. Evidence of enhanced sympathetic nervous activity in patients with decompensated cirrhosis. Gut 1984;25(10):1034–1043.
[33] Tage-Jensen U, Henriksen JH, Christensen E, et al. Plasma catecholamine level and portal venous pressure as guides to prognosis in patients with cirrhosis. J Hepatol 1988;6(3):350–358.
[34] Mookerjee RP. Acute-on-chronic liver failure: the liver and portal haemodynamics. Curr Opin Crit Care 2011;17(2):170–176.
[35] Rockey DC. Hepatic fibrosis, stellate cells, and portal hypertension. Clin Liver Dis 2006;10(3):459–479 [vii-viii].
[36] Hendrickson H, Chatterjee S, Cao S, et al. Influence of caveolin on constitutively activated recombinant eNOS: insights into eNOS dysfunction in BDL rat liver. Am J Physiol Gastrointest Liver Physiol 2003;285(3):G652–G660.
[37] Mookerjee RP, Malaki M, Davies NA, et al. Increasing dimethylarginine levels are associated with adverse clinical outcome in severe alcoholic hepatitis. Hepatology 2007;45(1):62–71.
[38] Mookerjee RP, Sen S, Davies NA, et al. Tumour necrosis factor alpha is an important mediator of portal and systemic haemodynamic derangements in alcoholic hepatitis. Gut 2003;52(8):1182–1187.
[39] Mookerjee RP, Dalton RN, Davies NA, et al. Inflammation is an important determinant of levels of the endogenous nitric oxide synthase inhibitor asymmetric dimethylarginine (ADMA) in acute liver failure. Liver Transpl 2007;13(3):400–405.
[40] McConnell M, Iwakiri Y. Biology of portal hypertension. Hepatol Int 2018;12(Suppl 1):11–23.
[41] Mendez-Sanchez N, Valencia-Rodriguez A, Coronel-Castillo C, et al. The cellular pathways of liver fibrosis in non-alcoholic steatohepatitis. Ann Transl Med 2020;8(6):400.
[42] Weiskirchen R, Tacke F. Cellular and molecular functions of hepatic stellate cells in inflammatory responses and liver immunology. Hepatobiliary Surg Nutr 2014;3(6):344–363.
[43] Liu TT, Ding TL, Ma Y, et al. Selective alpha1B- and alpha1D-adrenoceptor antagonists suppress noradrenaline-induced activation, proliferation and ECM secretion of rat hepatic stellate cells in vitro. Acta Pharmacol Sin 2014;35(11):1385–1392.
[44] Dubuisson L, Desmouliere A, Decourt B, et al. Inhibition of rat liver fibrogenesis through noradrenergic antagonism. Hepatology 2002;35(2):325–331.
[45] Ziolkowski N, Grover AK. Functional linkage as a direction for studies in oxidative stress: alpha-adrenergic receptors. Can J Physiol Pharmacol 2010;88(3):220–232.
[46] Wang B, Wang Y, Wu Q, et al. Effects of alpha2A adrenoceptors on norepinephrine secretion from the locus coeruleus during chronic stress-induced depression. Front Neurosci 2017;11:243.
[47] Tacke F. Targeting hepatic macrophages to treat liver diseases. J Hepatol 2017;66(6):1300–1312.
[48] Mookerjee RP, Lackner C, Stauber R, et al. The role of liver biopsy in the diagnosis and prognosis of patients with acute deterioration of alcoholic cirrhosis. J Hepatol 2011;55(5):1103–1111.
[49] Trevisani F, Sica G, Mainqua P, et al. Autonomic dysfunction and hyperdynamic circulation in cirrhosis with ascites. Hepatology 1999;30(6):1387–1392.
[50] Hansen HH, Feigh M, Veidal SS, et al. Mouse models of nonalcoholic steatohepatitis in preclinical drug development. Drug Discov Today 2017;22(11):1707–1718.

Keywords: Sympathetic signalling; cirrhosis; metabolic dysfunction-associated steatotic liver disease; steatohepatitis; noradrenaline; portal pressure.
Received 2 March 2025; received in revised form 21 May 2026; accepted 2 June 2026; Available online 9 June 2026

Journal of Hepatology, Volume *8*

Supplemental information

Alpha 2A adrenergic receptor antagonism reduces fibrosis, inflammation and portal hypertension in models of chronic liver disease

Karen Louise Thomsen, Vikram Sharma, Helen Jones, Anne Catrine Daugaard Mikkelsen, Nathan Davies, Abeba Habtesion, Sara Ellegaard Paaske, Jane Macnaughtan, Maria Castanho Martins, Rocio Gallego-Durán, Manuel Romero-Gómez, Stephen Hamilton-Dutoit, Krista Rombouts, Rajiv Jalan, and Rajeshwar Prosad Mookerjee

Alpha 2A adrenergic receptor antagonism reduces fibrosis, inflammation and portal hypertension in models of chronic liver disease

Karen Louise Thomsen, Vikram Sharma, Helen Jones, Anne Catrine Daugaard Mikkelsen, Nathan Davies, Abeba Habtesion, Sara Ellegaard Paaske, Jane Macnaughtan, Maria Castanho Martins, Rocio Gallego-Durán, Manuel Romero-Gómez, Stephen Hamilton-Dutoit, Krista Rombouts, Rajiv Jalan, Rajeshwar Prosad Mookerjee

Table of contents

Supplementary materials and methods……………………………………………………2

Table S1………………………………………………………………………………..14

Supplementary references……………………………………………………………...15

SUPPLEMENTAL MATERIALS AND METHODS

Animals

Male Sprague Dawley rats (body weight 220-300 g; Charles River, UK) were housed in a temperature and light controlled (12 hours light/dark cycle) facility at University College London. All animals were allowed to acclimatise for a week followed by randomization. All procedures were performed in accordance with UK Home Office Animals (Scientific Procedures) Act 1986 Amendment Regulations 2012, following approval from the local animal welfare and ethical review board. The animal experiments are reported in accordance with the ARRIVE guidelines (1).

BDL animal model: Rats underwent bile duct ligation (BDL) as described previously (2, 3), whilst sham-operated rats had a laparotomy without BDL (4). Four weeks after BDL or sham operation, rats were allocated either: (i) Sham-operated (control) rats administered subcutaneous (sc) saline BD, (ii) BDL rats administered sc saline BD or (iii) BDL rats receiving sc injection of the selective ADRA2a antagonist BRL44408 (Sigma-Aldrich Company Ltd., Dorset, UK) (10 mg/kg) BD within 24 hours prior to haemodynamic measurements. Also, sham-operated rats administered sc BRL44408 BD were included for haemodynamic measurements. Similarly, a cohort of BDL rats were also administered Yohimbine hydrochloride (YoHCl) (Sigma-Aldrich) (0.4mg/kg) for 2 doses, within 24 hours of haemodynamic assessment.

For all groups, animals were anaesthetized (induction 5% Isoflurane in oxygen, maintenance 1% in room air) and a cannula placed in the left carotid artery for continuous mean arterial pressure (MAP) monitoring (Biopac US). Following a laparotomy, a sub-group of animals underwent portal and hepatic arterial blood flow measurement (Transonic US). The portal pressure was

measured at the end of the procedure by inserting a 25G needle attached to a pressure transducer directly into the portal vein and values recorded after a period of stabilization, as we have shown previously (5).

Metabolic dysfunction-associated steatohepatitis (MASH) animal model: Twenty-four rats were fed normal chow (NC) (RM1; Special Diet Services, Witham, UK) or a high-fat, high-cholesterol (HFHC) diet (D09052204Y; Research Diets, USA) for 16 weeks as described previously (6, 7). The standard diet was composed of the following energy sources: 67 g carbohydrates (70 kcal/100 kcal), 4 g fat (10 kcal/100 kcal), and 19 g protein (20 kcal/100 kcal) per 100 g diet. The HFHC diet contained 19 g carbohydrates (15 kcal/100 kcal), 39 g fat (65 kcal/100 kcal), 2 g cholesterol, and 27 g protein (20 kcal/100 kcal) per 100 g diet. The fat was mainly from cocoa butter. The diets were provided *ad libitum* and the daily leftovers were weighed to determine the caloric intake. For the final 8 weeks of the study, HFHC fed rats were randomly allocated to receive YoHCl (Sigma-Aldrich) in their drinking water or control. Fluid was adjusted to achieve an average dose of 0.4 mg/kg/day. The animal groups were: (i) NC group: NC for 16 weeks (n=9), (ii) HFHC group: HFHC diet for 16 weeks (n=6) and (iii) HFHC+YoHCl group: HFHC diet and YoHCl for a final 8 weeks of study (n=9).

All rats from both experimental protocols were exsanguinated under terminal anaesthesia; blood was withdrawn into heparin or EDTA vacutainers (BD Biosciences, Oxford, UK), centrifuged (3500 rpm, 10 min, 4°C), and plasma stored at -80°C until further analyses. Liver was weighed and tissue samples were snap frozen in liquid nitrogen and stored at −80°C or fixed in 10% neutral buffered formalin. All of the assays used in this study had been validated for use in rats.

Hepatic Stellate Cell (HSCs)

Cell isolation and culture

Primary human (h)HSCs were isolated according to Mederacke et al (8) with modifications for human liver (9) (REC reference 21/WA/0388). HSCs were isolated from normal human liver (i.e. without inflammation and fibrosis) (10), obtained after liver wedge section surgery in patients with primary liver cancer or liver metastasis from extrahepatic cancers (9). Briefly, 10 g of total human liver tissue was digested with 0.01% Collagenase, 0.05% Pronase and 0.001% DNase I without performing perfusion. The homogenate was filtered through a 100m cell strainer (BD Biosciences) and the flow-through was centrifuged at 50g for 2 min at 4°C. After washing the supernatant, gradient centrifugation was performed at 1400g for 17 min at 4°C using an 11.5% Optiprep gradient (Sigma-Aldrich). Finally, the interface was collected and washed. Purity of hHSC was established by detection of CD140b (PDGFRbeta), CD29 (Integrin beta 1) and Cytoglobin B (CYGB).

Cells were cultured in complete medium (CM) containing Iscove's Modified DMEM (IMDM, Thermo Fisher Scientific, Watlham, USA), supplemented with 20% foetal bovine serum (FBS, Thermo Fisher Scientific), 2 mM Glutamine, 1X non-essential amino acids, 1.0 mM sodium pyruvate, 1X antibiotic-antimycotic (Life Technologies, Carlsbard, USA), and maintained under standard conditions in a humidified incubator under 5% CO2 in air at 37°C. Medium was refreshed twice a week and cells were passaged when subconfluent with 1X Trypsin (Gibco, Thermo Fisher Scientific) (9). Rat HSCs cells were a kind gift from Jonathan Fallowfield (University of Edinburgh). Rat HSCs were cultured in DMEM (high glucose, with sodium pyruvate & Glutamax) with 10% FBS (Life Technologies).

Treatments

All treatments with agonist or antagonist were performed in serum free medium (SFM). Human HSCs were seeded at 300,000 cells per well in CM in 6-well plates. After 24 hours CM was replaced with SFM for a further 24 hours to induce quiescence. Cells were subsequently treated with SFM, 1% FBS-CM (CM with FBS reduced to 1%), CM, or SFM + 5uM guanfacine hydrochloride (CAS: 29110-48-3; Sigma-Aldrich) for 24 hours.

Gel contraction

Human and rat HSCs were cultured within 3D collagen gels as previously described (11). Twenty four well cell culture plates were pre-coated with sterile 2% BSA (Sigma-Aldrich) in phosphate buffered saline (PBS). Ice-cold neutral collagen solution was made by mixing 1 part 0.2M HEPES pH 8.0 (Life Technologies), 4 parts >2mg/ml rat tail collagen type I (First Link (UK) Ltd., Wolverhampton, UK), and 5 parts SFM. Equal volumes of the neutral collagen solution and HSCs resuspended at 80.000 cells/ml in SFM or SFM with 5uM BRL44408 (CAS: 118343-19-4; Tocris Bioscience, Abingdon, UK) were mixed. One ml was added to each well and allowed to polymerise for ≥2 hours at 37°C 5% CO_2. After polymerisation, 1ml of SFM, SFM with 5uM guanfacine, SFM with 50uM guanfacine, or SFM with 5uM guanfacine and 5uM BRL44408 was added causing detachment of the gel from the plate. After 6 hours the contracted gels were imaged using a Fluorchem M imager (ProteinSimple) with white light transillumination and 607/36 nm filter. Gel areas were measured using ImageJ v1.49 software (12).

RT-qPCR of primary human HSCs

In hHSCs, mRNA expression of ADRA2A and PDGFR-β were determined by RT-qPCR. RNA was extracted from cell pellets of 14 donors using the Rneasy Mini kit (Qiagen Ltd., Manchester, UK). RNA concentration was measured using the Qubit RNA Broad Range assay (Invitrogen, UK). A total amount of 800ng of each sample were reverse transcribed using the High-Capacity cDNA Reverse Transcription Kit (Thermo Fisher Scientific) according to manufacturer's instructions. qPCR was performed using TaqMan Fast Advanced Master Mix (Thermo Fisher) with Assays-on-Demand primers (Thermo Fisher) on a QuantStudio 7 Pro (Applied Biosystems). Data was analysed using the delta Ct method with GAPDH as endogenous control.

Metabolic dysfunction-associated steatotic liver disease (MASLD) patients

Fifteen MASLD patients were recruited from the Hepatology Unit of Virgen del Rocío University Hospital, Spain. Patients were ≥18 years of age and MASLD was diagnosed based on the following inclusion criteria: a) ultrasonographic evidence of hepatic steatosis; b) elevated liver enzymes; c) metabolic syndrome following ATPIII criteria (13). Exclusion criteria were as follows: significant alcohol intake (≥30 g/day in men and ≥20 g/day in women), recreational drugs abuse, and evidence of viral or autoimmune hepatitis, HIV, drug-induced fatty liver or other metabolic liver diseases. Study procedures agreed with the ethical standards of the responsible committee on human experimentation and were approved by the human research ethics committee (C.I. 0359-N-15). Informed consent was ontained from all participants.

Percutaneous liver biopsies were obtained after an overnight fast and performed under local anaesthesia and ultrasound guidance using a tru-cut needle. The biopsy samples were divided in two and immediately snap-frozen and stored at -80ºC or formalin-fixed overnight and paraffin-embedded. The liver specimens were stained with haematoxylin-eosin, reticulin and Masson´s

trichrome. An experienced pathologist unaware of clinical data assessed the samples to determine the Non-alcoholic fatty liver disease (NAFLD) Activity Score (NAS) and fibrosis stage by Kleiner Score (14).

Messenger RNA expression of ADRA2a was evaluated using RT-qPCR. Total RNA was isolated from the snap-frozen liver specimens using a mirVana Isolation kit (Life Technologies) following manufacturer´s instructions. RNA Integrity number (RIN) was measured with an Agilent 2100 Bioanalyzer System, and RNA concentration was evaluated using a Nanodrop One (Thermo Fisher Scientific). Reverse transcription was performed on 500 ng per patient sample using the PrimeScript RT Master Mix (Takara, Shiga, Japan). qPCR was performed as described for the animal studies.

Blood analyses

Monocyte Phagocytic Studies

Whole blood preparation: 2 ml of blood was collected from the portal vein. 40 ml of RBC lysis buffer was added to each 2 ml sample, vortexed and incubated for 15 min at room temperature. Following centrifugation at 450g for 5 min, the supernatant was discarded, and the pellet re-suspended with 1 ml of complete culture media (RPMI (Gibco), 10% FBS, 200mM L-Glutamine). The cell number was assessed using the nucleoCounter method and adjusted to $1x10^7$ cell/ml.

The cells were centrifuged at 500g for 5 min at 4ºC and the supernatant discarded. 200 µl media containing latex beads (L2778, Sigma) were added to the pellet and incubated at 37°C in the dark for 20 min. 5 ml of ice-cold PBS was then added and centrifuged at 800g for 5 min at 4ºC. The pellet was then washed with 5 ml of cold PBS and centrifuged. Cells were incubated with anti-

CD32 antibody for 10 min at 4°C followed by incubation with PE-conjugated anti-CD11b (MCA275A647 (Clone OX-42), Abd Serotec) and A647-conjugated anti-CD43 (202810 (Clone W3/13), Biolegend) or isotype control for 30 min at 4°C in the dark. The cells were then washed with 1ml of FACS buffer, centrifuged and re-suspended in 100 µl FACS buffer solution. Samples were kept at 4°C and analysed immediately using a FACS LSR II Flow Cytometer (Becton Dickinson). Data was analysed using FlowJo software.

Liver tissue analyses

Collagen proportionate area (CPA) measurement

CPA was determined on NanoZoomer images of PSR stained whole sections of liver. Images were captured at 20x magnification and analysis was performed on 5x magnification TIFF images extracted from the NDPI files using the NDPI-split tool (15). All subsequent digital image analysis was performed using ImageJ2 (16, 17). A region of interest (ROI) covering the entire liver section excluding only cutting artifacts and lumen of large vessels was selected for analysis. A custom macro script was written to measure the total area of the ROI, threshold the image to segregate only the red-stained collagen and measure the area of the thresholded portion of the ROI. The script was run in batch mode for all the images analysed. CPA was calculated as the thresholded ROI area as a percentage of the total ROI area.

Western blot analysis

In BDL animals, protein expression of ADRA2a, caveolin-1, eNOS and p-eNOS was measured using WB analysis as described previously (11). Proteins were isolated from fresh-frozen liver tissues, which were homogenized in lysis buffer containing 50 mM Tris-HCl (pH 7.6) with a

protease inhibitor cocktail (Sigma-Aldrich), protein methyl sulphonyl fluride (PMSF) and ethylene diamine tetra acetic acid (EDTA) using a tissue homogenizer. The lysates were centrifuged for 60 min at 10,000 rpm and the supernatants measured for protein concentration by the Bradford method. Extracts containing equal amounts of protein was denatured and separated on 4-12% NuPAGE Bis-Tris Gels and blotted on to PVDF membranes (Invitrogen). The membranes were then incubated with different primary antibodies, and specific protein bands detected using HRP-conjugated secondary antibodies with an enhanced ECL detection kit, rabbit anti-α tubulin (1:1000; Upstate, Biotechnology, N.Y.) serving as a loading control.

RNA isolation and RT-qPCR

In MASH animals, mRNA expression of ADRA2A and TIMP metallopeptidase inhibitor 1 (TIMP1) was determined using RT-qPCR. Frozen liver was homogenized in TRI reagent (Sigma-Aldrich) using 7mm steel beads with a TissueLyser LT (Qiagen Ltd.). After extraction with chloroform total RNA was isolated using a RNeasy mini kit (Qiagen Ltd.). RNA concentration was measured using a nanodrop (Labtech International Ltd., Uckfield, UK). Reverse transcription was performed on 1ug RNA per sample with combined polyT and random hexamer primers using a Quantitect reverse transcription kit (Qiagen Ltd.). A no-RT control reaction omitting the reverse transcriptase enzyme was also performed. qPCR was performed using hydrolysis probe assays (Integrated DNA Technologies, BVBA., Leuven, Belgium) and Rotor-gene probe PCR mix (Qiagen Ltd.) on a Rotor-gene 6000 (Qiagen Ltd.). Data was analysed by calculating relative quantity (RQ; $2^{\Delta ct}$) using the average of the NC group as the calibrator. RQ was normalised using a normalisation factor generated from the geometric mean of RQ of 3 reference genes (Tbp, Ppia, Hmbs) (18).

Hepatic MMP-2 protein expression

In MASH animals, protein expression of active MMP-2 was measured using gel zymography. Frozen liver was homogenised in RIPA buffer (Pierce Biotennchnology Inc., Rockford IL, USA) with complete ultra mini EDTA-free protease inhibitors and phoSTOP phosphatase inhibitors (Roche Diagnostics Ltd.) using 7mm steel beads with a TissueLyser LT (Qiagen Ltd.). Protein concentration was measured using a BCA assay kit (Pierce Biotechnology Inc.). Equal amounts of protein were separated on 10% Novex Tris-Glycine gels containing 0.1% gelatine under non-reduced denaturing conditions using tris-glycine-SDS running buffer (Life Technologies). The molecular weight markers were Novex Sharp prestained standard (Life Technologies). Following electrophoresis, the gels were incubated in zymogram renaturing buffer (Life Technologies) and then developed overnight at 37 °C in zymogram developing buffer (Life Technologies). The developed gels were stained overnight in an excess of GelCode Blue Safe stain (Sigma-Aldrich) and imaged using a UVP VioSpectrum imaging system (UVP Ltd, Cambridge, UK). Densitometry was measured using ImageJ2 (16, 17).

Hepatic protein expression of chemokines

Hepatic protein expression of the selected chemokines C-C motif ligand 3 (Ccl3), C-X3-C motif ligand 1 (Cx3cl1) = fractalkine and C-X-C motif chemokine 5 (Cxcl5) was then assessed by ELISA. Frozen liver was homogenised in lysis buffer containing sodium deoxycholate (RABLYSIS1; Sigma-Aldrich) with protease phosphatase inhibitors as above. Protein concentration was measured by BCA. Sandwich ELISAs were used to measure Ccl3 (20ug protein per well; Cloud-Clone Corp, Katy, TX, USA), Cx3Cl1 (20ug protein per well; RAB0115; Sigma-

Aldrich), and Cxcl5 (100ug protein per well; ERCXCL5CL; Life Technologies) according to the manufacturers' instructions in a 96 well plate (as above). .

Hepatic CD68 protein expression

In MASH animals, 3-5mm liver sections were dewaxed in xylene and rehydrated through graded alcohols prior to immunohistochemical (IHC) detection of CD68. Heat-induced epitope retrieval was performed by boiling for 20mins in 10mM sodium citrate pH 6 in a microwave. Blocking steps consisted of avidin/biotin blocking (Vector Laboratories Ltd., Peterborough, UK), general protein blocking (2.5% normal horse serum; Vector Laboratories Ltd.), and peroxidase blocking (3% H_2O_2). Blocked sections were incubated overnight at 4°C in biotinylated mouse anti-CD68 (1:100 dilution). Secondary detection was performed using HRP-conjugated ABC reagent (VectaStain Elite; Vector Laboratories Ltd.) and DAB substrate (Vector Laboratories Ltd.). Stained sections were counterstained using Gills haematoxylin (Sigma-Aldrich) and mounted in DPX. Slides were digitised and quantified as above (15). All subsequent digital image analysis was performed using ImageJ2 (16, 17). A ROI covering the entire liver section excluding only cutting artefacts and lumen of large vessels was selected for analysis. A custom macro script was written to measure the total area of the ROI, perform colour deconvolution to separate the DAB stain from the haemotoxylin counter-stain, threshold the image to segregate only the DAB-stained regions, and measure the area of the thresholded portion of the ROI. The script was run in batch mode for all the images analysed. IHC-stained area was calculated for the thresholded ROI area as a percentage of the total ROI area.

Kupffer cell phagocytic function and ROS production

Isolation of non-parenchymal cell fraction from rodent liver tissue:

The liver was perfused with 5 ml of ice-cold PBS and homogenized in Hanks balanced salt solution (HBSS) (Ca^{2+} and Mg^{2+}) with collagenase (0.01%) and DNase I (0.001%) (Gibco). The homogenate was transferred to a 50 ml Falcon tube and incubated at 37°C for 20 min prior to filtration through a 100 µm cell strainer. This was then centrifuged at 50g for 2 min at 4°C and the supernatant then centrifuged at 450g for 10 min at 4°C. The supernatant was discarded, and the pellet re-suspended in HBSS (no Ca^{2+} and Mg^{2+}), DNase I 0.01%, bovine serum albumin (0.25%) and centrifuged at 450g for 10 min at 4°C. The pellet was then re-suspended in 3.9 ml of RPMI 1640 and mixed gently with 2.1 ml of RPMI (Thermo Fisher Scientific) and Optiprep 22% (Stemcell Technologies). RPMI was then layered on top followed by a 25-minute centrifugation step at 1500g without brake at 4°C. The non-parenchymal cells were isolated from the interface, re-suspended in an equivalent volume of HBSS (free Ca^{2+} and Mg^{2+}), DNase I 0.01%, bovine serum albumin (0.25%) and centrifuged at 800g at 4°C for 10 min. The pellet was re-suspended in 5 ml of Red Cell Lysis Buffer (BioLegend) and incubated for 5 min at 4°C with occasional shaking. The reaction was then stopped by addition of 10 ml of PBS. This was then centrifuged at 800g for 10 min at 4ºC, the supernatant discarded, and the cells re-suspended in 3ml of culture media. The cells were counted and adjusted to a concentration of 10^7 cells/ml. $1x10^6$ cells were used in all subsequent assays.

Kupffer cell population studies:

2 µl anti-CD32 antibody (550270 (Clone D34-485), Becton Dickinson) was added to 10^6 cells (non-parenchymal cell fraction) and incubated for 5 min at 4°C. The cells were then co-incubated with anti-CD163 antibody (MCA342A647 (Clone ED2), AbD Serotec) and anti-CD68 antibody

(MCA341B, (Clone ED1), Abd Serotec) or isotype controls for 30 min at 4°C in the dark. The cells were washed with 1 ml of FACS buffer (42020, Cell staining buffer, BioLegend), centrifuged and re-suspended in 100 µl FACS buffer solution and analysed immediately on a Becton Dickinson LSR II flow cytometer. Data was analysed using FlowJo software.

Kupffer cell ROS studies:

20 µg/ml of E.coli endotoxin was added to 1x 10^6 non-parenchymal cells sample and incubated for 30 min at 37°C. ROS inducer at a final concentration of 200-500uM was used as a positive control. The samples were then centrifuged at 500g for 5 min and the supernatant discarded. The cells were re-suspended in 5 ml of wash buffer, centrifuged at 500g for 5 min and the supernatant removed. The cells were re-suspended in 500 µl of ROS detection solution and incubated for 30 min at 37°C in the dark. Following centrifugation, the cells were re-suspended in 100 µl of FACS buffer, and incubated with anti-CD32 antibody (550270 (Clone D34-485) for 10 min at 4°C. Cells were then incubated with anti-CD163 antibody or isotype control for 30 min at 4°C in the dark. The cells were then washed with 1 ml of FACS buffer, centrifuged and re-suspended in 100 µl FACS buffer solution. samples were kept at 4°C and analysed immediately using a FACS LSR II Flow Cytometer (Becton Dickinson). Data was analysed using FlowJo software.

Table S1.
Clinical and biochemical characteristics and histological scores

	F0 fibrosis	F1-3 fibrosis
Age (years)	42 ± 11	56 ± 12
Sex (m/f)	4/3	5/3
BMI	28.6 ± 7.1	32.7 ± 1.9
ALT (U/L)	33 ± 15	54 ± 30
AST (U/L)	28 ± 10	47 ± 18
HOMA	2.7 ± 1.6	4.0 ± 3.0
NAS score	1.5 ± 0.9	3.4 ± 1.1
Fibrosis score	0.0 ± 0.0	2.1 ± 0.9

Age, sex, body mass index (BMI) and plasma concentrations of alanine aminotransferase (ALT) and aspartate aminotransferase (AST), homeostatic model assessment (HOMA) and histological scores of liver tissue (non-alcoholic fatty liver disease (NAFLD) activity score (NAS) and fibrosis) in patients with metabolic dysfunction-associated steatotic liver disease and F0 (n=7) and F1-3 (n=8) fibrosis.

Supplementary references

1. Percie du Sert N, Hurst V, Ahluwalia A, Alam S, Avey MT, Baker M, et al. The ARRIVE guidelines 2.0: Updated guidelines for reporting animal research. PLoS Biol. 2020;18(7):e3000410.
2. Kountouras J, Billing BH, Scheuer PJ. Prolonged bile duct obstruction: a new experimental model for cirrhosis in the rat. Br J Exp Pathol. 1984;65(3):305-11.
3. Poo JL, Estanes A, Pedraza-Chaverri J, Cruz C, Perez C, Huberman A, et al. [Chronology of portal hypertension, decreased sodium excretion, and activation of the renin-angiotensin system in experimental biliary cirrhosis]. Rev Invest Clin. 1997;49(1):15-23.
4. Harry D, Anand R, Holt S, Davies S, Marley R, Fernando B, et al. Increased sensitivity to endotoxemia in the bile duct-ligated cirrhotic Rat. Hepatology. 1999;30(5):1198-205.
5. Mookerjee RP, Mehta G, Balasubramaniyan V, Mohamed Fel Z, Davies N, Sharma V, et al. Hepatic dimethylarginine-dimethylaminohydrolase1 is reduced in cirrhosis and is a target for therapy in portal hypertension. J Hepatol. 2015;62(2):325-31.
6. Thomsen KL, Gronbaek H, Glavind E, Hebbard L, Jessen N, Clouston A, et al. Experimental nonalcoholic steatohepatitis compromises ureagenesis, an essential hepatic metabolic function. Am J Physiol Gastrointest Liver Physiol. 2014;307(3):G295-301.
7. De Chiara F, Thomsen KL, Habtesion A, Jones H, Davies N, Gracia-Sancho J, et al. Ammonia Scavenging Prevents Progression of Fibrosis in Experimental Nonalcoholic Fatty Liver Disease. Hepatology. 2020;71(3):874-92.
8. Mederacke I, Dapito DH, Affo S, Uchinami H, Schwabe RF. High-yield and high-purity isolation of hepatic stellate cells from normal and fibrotic mouse livers. Nat Protoc. 2015;10(2):305-15.
9. Rombouts K, Carloni V. Determination and Characterization of Tetraspanin-Associated Phosphoinositide-4 Kinases in Primary and Neoplastic Liver Cells. Methods Mol Biol. 2016;1376:203-12.
10. Caon E, Martins M, Hodgetts H, Blanken L, Vilia MG, Levi A, et al. Exploring the impact of the PNPLA3 I148M variant on primary human hepatic stellate cells using 3D extracellular matrix models. J Hepatol. 2024;80(6):941-56.
11. Jalan R, De Chiara F, Balasubramaniyan V, Andreola F, Khetan V, Malago M, et al. Ammonia produces pathological changes in human hepatic stellate cells and is a target for therapy of portal hypertension. J Hepatol. 2016;64(4):823-33.
12. Schneider CA, Rasband WS, Eliceiri KW. NIH Image to ImageJ: 25 years of image analysis. Nature Methods. 2012;9(7):671-5.
13. Expert Panel on Detection E, Treatment of High Blood Cholesterol in A. Executive Summary of The Third Report of The National Cholesterol Education Program (NCEP) Expert Panel on Detection, Evaluation, And Treatment of High Blood Cholesterol In Adults (Adult Treatment Panel III). JAMA. 2001;285(19):2486-97.
14. Kleiner DE, Brunt EM, Van Natta M, Behling C, Contos MJ, Cummings OW, et al. Design and validation of a histological scoring system for nonalcoholic fatty liver disease. Hepatology. 2005;41(6):1313-21.
15. Deroulers C, Ameisen D, Badoual M, Gerin C, Granier A, Lartaud M. Analyzing huge pathology images with open source software. Diagn Pathol. 2013;8:92.
16. Schindelin J, Arganda-Carreras I, Frise E, Kaynig V, Longair M, Pietzsch T, et al. Fiji: an open-source platform for biological-image analysis. Nat Methods. 2012;9(7):676-82.
17. Rueden CT, Schindelin J, Hiner MC, DeZonia BE, Walter AE, Arena ET, et al. ImageJ2: ImageJ for the next generation of scientific image data. BMC Bioinformatics. 2017;18(1):529.
18. Hellemans J, Mortier G, De Paepe A, Speleman F, Vandesompele J. qBase relative quantification framework and software for management and automated analysis of real-time quantitative PCR data. Genome Biol. 2007;8(2):R19.
